# OLFML3 Promotes IRG1 Mitochondrial Localization and Modulates Mitochondrial Function in Macrophages

**DOI:** 10.7150/ijbs.103859

**Published:** 2025-02-26

**Authors:** Qijun Yu, Hong Mei, Qian Gu, Ran Zeng, Yanan Li, Junjie Zhang, Chenxu Gao, Hai Fang, Jieming Qu, Jia Liu

**Affiliations:** 1 Department of Pulmonary and Critical Care Medicine, Ruijin Hospital, Institute of Respiratory Diseases, Shanghai Jiao Tong University School of Medicine, Shanghai 200025, China; and Shanghai Key Laboratory of Emergency Prevention, Diagnosis and Treatment of Respiratory Infectious Diseases, Shanghai 200025, China.; 2 Shanghai Institute for Advanced Immunochemical Studies and School of Life Science and Technology, ShanghaiTech University, Shanghai 201210, China.; 3 Shanghai Institute of Hematology, State Key Laboratory of Medical Genomics, National Research Center for Translational Medicine at Shanghai, Ruijin Hospital, Shanghai Jiao Tong University School of Medicine, Shanghai 200025, China.; 4 Guangzhou Laboratory, Guangzhou International Bio Island, No. 9 XingDaoHuanBei Road, Guangzhou, 510005, Guangdong, China.; 5 Shanghai Clinical Research and Trial Center, Shanghai, 201210, China.

**Keywords:** acute Lung Injury, IRG1, macrophage, mitochondria, OLFML3

## Abstract

Olfactomedin-like protein 3 (OLFML3), belonging to olfactomedin (OLF) protein family, has poorly defined functions. Recent studies have reported the functions of OLFML3 in anti-viral immunity and tumorigenesis. In this study, we investigated the roles of OLFML3 in macrophages. In LPS- or *Pseudomonas aeruginosa*-induced acute lung injury (ALI) mouse model, OLFML3 depletion exacerbated inflammatory response, leading to reduced survival. OLFML3 achieved the *in vivo* activity by regulating macrophage phagocytosis and migration. Mass spectrometry analysis revealed immunoresponsive gene 1 (IRG1) as an OLFML3-interacting protein. IRG1 is a mitochondrial decarboxylase that catalyzes the conversion of *cis*-aconitate to itaconate, a myeloid-borne mitochondrial metabolite with immunomodulatory activities. Further investigation showed that OLFML3 could prevent LPS-induced mitochondrial dysfunction in macrophages by maintaining the homeostasis of mitochondrial membrane potential (MMP), mitochondrial reactive oxygen species (mtROS) and itaconate-related metabolites. In-depth protein-protein interaction studies showed that OLFML3 could promote IRG1 mitochondrial localization via a mitochondrial transport protein, apoptosis inducing factor mitochondria associated 1 (AIFM1). In summary, our study showed that OLFML3 could facilitate IRG1 mitochondrial localization and prevent LPS-induced mitochondrial dysfunction in macrophages.

## Introduction

Olfactomedin-like protein 3 (OLFML3) is a 406-amino acid glycoprotein. OLFML3 is also recognized as hOLF44 1 and HNOEL-iso 2 in human, ONT3 in mice 3 and ONT1 in chicken 4 and *Xenopus* 5. OLFML3 is a member of the olfactomedin (OLF) protein family which contains over 200 members present in diverse species ranging from *Caenorhabditis elegans* to *Homo sapiens* 6. A recent phylogenetic study has defined OLF family as nine evolutionarily distinct subfamilies, and OLFML3 resides in subfamily VII 6. Structurally, OLFML3 and other members in subfamily VII are characterized with an N-terminal coiled-coil region (CC domain) and a C-terminal OLF-like domain (OLF domain) 6.

Human *Olfml3* gene is located in band p13.1 in chromosome 1 and contains three exons and two introns spanning a 2.9 Kbp region. Human *Olfml3* gene encodes a 1,852-nucleotide mRNA, composed of a 1,221-nucleotide coding DNA sequence (CDS) and two flanking untranslated region (UTRs) 1. OLFML3 mRNA is differentially expressed in multiple human tissues, where placenta shows high expression and leukocytes show little expression 1. Interestingly, it has been also reported that during tumor progression, OLFML3 expression is tightly regulated at the transcriptional level 7-9 and that viral infection induced the expression of OLFML3 10.

The exact functions and mechanisms of action of OLFML3 are poorly understood and seem to vary among species. In *Xenopus*, OLFML3 (ONT1) is indispensable in bone morphogenic protein (BMP) signaling for axial development. *Xenopus* ONT1 acts as a scaffold to bind chordin and BMP1/Tolloid-class proteinase (B1TP) through OLF and CC domains respectively 5. The function of OLFML3 in embryonic development was also reported in pigs 11. In mouse, however, OLFML3 (ONT3) knockout in CD-1 background led to normal and fertile offsprings 3, suggesting a dispensable role of OLFML3. Additionally, human and rat OLFML3 have distinct expression patterns with the former being widely expressed and the latter specifically expressed in brain and ovary 6.

Recent studies have highlighted the functions of OLFML3 in human diseases. OLFML3 was identified as a signature gene in microglia 12-14 and subsequent studies revealed a tumorigenic function of OLFML3 in glioblastoma 7, 8, 14, 15. OLFML3 was also found to be involved in pericyte proliferation and function as pro-angiogenic molecule in tumor microenvironment 16, 17. Blockade of OLFML3 could inhibit tumor growth and angiogenesis in colorectal cancer and cholangiocarcinoma 18, 19. In addition, mutations in OLFML3 appear to be associated with eye diseases such as glaucoma 20, 21.

In our previous study, OLFML3 was identified as a suppressor protein to type I interferon (IFN) signaling during human rhinovirus (RV) infection 10. This prompted us to investigate a broader immunomodulatory function of OLFML3 in cellular response to pathogens or pathogen-associated molecular patterns (PAMPs). In the present study, we sought to explore the *in vivo* and *in vitro* function of OLFML3 during lipopolysaccharide (LPS) stimulation or bacterial infection. In-depth analyses uncovered a modulatory role of OLFML3 in macrophage phagocytosis and migration and in LPS-induced mitochondrial dysfunction. The modulatory activity of OLFML3 was achieved via interactions with immunoresponsive gene 1 (IRG1), an aconitate decarboxylase responsible for itaconate production. The interaction patterns between OLFML3 and IRG1 were examined in details.

## Materials and methods

### Mouse experiments

C57BL/6 *Olfml3^-/-^* mice were generated using CRISPR-Cas9 and provided by Gempharmatech Co (Nanjing, China). The mice were housed in environmentally controlled cages with a 12-h light-dark cycle and were provided with unrestricted access to food and water. Experimental male mice were selected between 8 to 10 weeks of age. BMDMs were isolated from 8-week-old male mice. For survival experiment, mice were intraperitoneally injected with 10 mg/kg LPS to induce sepsis (*n* = 11 per group). ALI was initiated by intranasal instillation of LPS (10 mg/kg) or PAO1 (2×10^6^ colony-forming units, CFU) (*n* = 5 or 6 per group).

For alveolar macrophage depletion experiment, 8-week C57BL/6 male mice were intranasally instilled with 50 μL clodronate liposomes (CLL, Cat# 40337ES08, Yeasen, Shanghai, China) twice. At 48 h after CLL treatment, 10 mg/kg LPS was intranasally administrated. Lung samples were harvested at 36 h post LPS treatment.

All animal procedures were performed in accordance with the ethical standards and approved by the Ethics Committee for Animal Experimentation at ShanghaiTech University (20230821001).

### Cell culture

RAW264.7 cells (Cat# CL-0190, Procell, Wuhan, China), HeLa cells (Cat# CRL-1958, American Type Culture Collection, ATCC), and HEK293T cells (Cat# SCSP-5209, Cell Bank of Shanghai Institutes for Biological Science, SIBS, Shanghai, China) were maintained in DMEM (Cat# D0819, Sigma-Aldrich, St. Louis, MO, USA) supplemented with 10% fetal bovine serum (FBS; Cat# 10091148, Gibco, Gaithersburg, MD, USA). L292 cells (Cat# CCL-1, ATCC) were cultured in IMDM (Cat# 12440053, Gibco) with 10% FBS. BMDMs were obtained from the tibia and fibula of 8-week-old male mice and cultured in IMDM supplemented with 10% FBS, 20% L929 cell supernatant, and 1% penicillin/streptomycin (Cat# 15140122, Gibco) for a period of 6 days.

### Generation of CRISPR-Cas9 knockout cells

Lentiviruses containing sgRNAs targeting *Olfml3* or *Aifm1* were produced in HEK293T cells and subsequently transduced into RAW264.7 cells. Following a 2-day incubation, single-cell clones were isolated using the MoFlo Astrios EQ Cell Sorter (Beckman Coulter, Brea, CA, USA). These clones were cultured for 7-10 days, and genomic DNA from each clone was extracted using the Quick Extraction kit (Cat# QE09050, Lucigen, Middleton, WI, USA). Sanger sequencing was then performed to identify and characterize mutations present at each allele. The sgRNA sequences can be found in Supplementary Data 3.

### Bacterial culture

The laboratory strain of *Pseudomonas aeruginosa* used in this study was PAO1 (Cat# ATCC-BAA-47; strain HER-1018). PAO1 was initially cultured on Luria Bertani (LB) agar plates (Cat# A507003, Sangon Biotech, Shanghai, China) and subsequently grown in 4 mL of LB broth for 12-16 h at 37 °C with agitation at 220 rpm. Then 0.1 mL of the PAO1 culture was diluted into 4 mL of fresh LB medium and incubated for 2.5 h until the optical density at 600 nm reached 0.5, corresponding to a concentration of 2 × 10^8^ colony-forming units per milliliter. Prior to *in vivo* nasal instillation, PAO1 was subjected to three washes with clean phosphate-buffered saline (PBS; Cat# MA0015-2, Meilunbio, Dalian, China) and then diluted in sterile PBS.

### Reverse transcription quantitative PCR (RT-qPCR)

The mRNA levels of OLFML3 and cytokines in RAW264.7 and BMDMs were analyzed by RT-qPCR assay. Total RNA was isolated from cells using Trizol (Cat# 15596018, Invitrogen, Camarillo, CA, USA) and then mRNA reverse transcribed into cDNA using PrimeScript™ RT reagent Kit with gDNA Eraser (Cat# RR047A, Takara, Dalian, China) following the instructions. RT-qPCR reaction in 3 μL contained 1×SYBR Green Mix (Cat# A25742, Applied Biosystems, Carlsbad, CA, USA), 2.5 μM of forward and reverse primers, 2 μL cDNA and nuclease-free water. RT-qPCR reaction was carried out in QuantStudio 6 Flex Real-Time PCR System (Applied Biosystems) using cycling conditions as previously described 10. Real-time fluorescence signals were monitored during each cycle, and quantitative data were collected and analyzed using QuantStudio software. The expression of each gene was normalized to reference genes as indicated.

### Western blotting

Whole-cell lysates were extracted from RAW264.7 or BMDMs using RIPA lysis buffer (Pierce) supplemented with protease inhibitors and a phosphatase inhibitor (Cat# P002, NCM Biotech, Suzhou, China). For detection of supernatant proteins, Minute High-Efficiency Protein Precipitation Kit (Cat# WA-006, Invert Biotechnologies, Plymouth, MN, USA) was employed to enrich the supernatant proteins using the traditional trichloroacetic acid (TCA) precipitation method. Cytoplasmic and nuclear proteins were separated using a nuclear protein extraction kit (Cat# R0050, Solarbio, Beijing, China) following supplier's instructions. The protein concentration in cell lysate were quantified using the BCA kit (Cat# P0010, Beyotime, Shanghai, China). Signals were detected using an ECL kit (Cat# MA0186, Meilunbio). The primary antibodies and dilution concentrations used in the western blotting experiments were as below: OLFML3 (1:250, Cat# MAB7324, R&D, Minneapolis, MN, USA), IRG1 (1:1000, Cat# ab222411, Abcam, Cambridge, MA, USA), Flag tag (1:1000, Cat# 14793S, Cat# 8146S, CST), HA-tag (1:1000, Cat# 3724S, CST), caspase-3 (1:1000, Cat# 9662S, CST), Tom20 (1:1000, Cat# ab186735, Abcam), COX IV(1:1000, Cat# ab202554, Abcam), β-tubulin (1:5000, Cat# 30302ES20, Yeasen), Lamin A/C (1;1000, Cat# 2032S, CST), Strep tag II (1:2000, Cat# A02230, Abbkine, Wuhan, China), AIFM1 (1:1000, Cat# 5318S, CST) and β-ACTIN (1:5000, Cat# 66009-1, Proteintech, Wuhan, China).

### Enzyme-linked immunosorbent assay (ELISA)

ELISA was used to quantify the level of MCP-1 in the cell supernatant and IL-1β, IL-6, TNF-α levels in bronchoalveolar lavage fluid (BALF) and serum following the manufacturer's guidelines (DAKEWE, Shenzhen, China). The absorbance was measured using an ELISA reader (CANY, Shanghai, China) at 450 nm, using 610 nm as a reference wavelength.

### RNA-seq

The wild-type and *Olfml3* knockout RAW264.7 cells and BMDMs were stimulated with 100 ng/mL LPS for 4 h. The RNA was prepared using Trizol. Then Samples were processed and sequenced by GENEWIZ company (Suzhou, China). Briefly, poly(A) mRNA was isolated using oligo (dT) beads and fragmentated using divalent cations and elevated temperatures. Random primers were employed for priming, enabling the synthesis of cDNA. Double-stranded cDNA was purified and then subjected to a comprehensive treatment, involving end repair and dA-tailing in a single reaction, followed by T-A ligation to attach adaptors to both ends. Size selection was performed on the adaptor-ligated DNA using DNA Clean Beads. PCR amplification was conducted using P5 and P7 primers and then validated for product quality. Libraries with distinct indices were multiplexed and loaded onto an Illumina HiSeq, Illumina Novaseq, or MGI2000 for sequencing. The sequencing was performed in a 2×150 paired-end (PE) configuration, following the manufacturer's guidelines.

Differentially expressed genes (DEGs) between knockout and wild type were identified using the limma R package (version 3.6.0) 22. Genes were considered significantly differentially expressed if they met the criteria of adjusted *p* value less than 0.05 and absolute fold change value no less than 1.5. *P* values were adjusted using the Benjamini-Hochberg method 23.

The R package SupraHex (version 1.42.0) 24, 25 was employed for clustering all DEGs and analyzing of sample correlation. In brief, a supra-hexagonal map consisting of 91 hexagons was trained using the self-organizing learning algorithm. Each hexagon contained co-clustered genes displaying similar expression patterns in response to *Olfml3* knockout. Hexagons with similar fold change values were clustered in close proximity. These supra-hexagonal maps presented sample-specific gene expression profiles. The raw data for generating the maps can be found in Supplementary Data 1.

Gene-centric analysis was performed using the R package XGR (version 1.1.9) 26, with a specific focus on GO Biological Process (GOBP) terms. This analysis involved a total of 28,555 genes that were annotated to GOBP terms. Enrichment p-values were computed using a one-sided Fisher's exact test and subsequently adjusted utilizing the Benjamini-Hochberg 23 method to account for multiple testing. During the analysis, the size of gene sets annotated by GOBP terms was set with a minimum of 5 and a maximum of 1,500. A filter for GOBP terms with an adjusted *p* value threshold of less than 0.05 was set. These criteria ensured that only terms with a minimum of 5 overlapping members from the input data were included.

The raw data can be found in the Supplementary Source Data file.

### Co-immunoprecipitation assay

Cells were lysed using ice-cold IP lysis buffer (Cat# A36797, Invitrogen) supplemented with a protease inhibitor cocktail (NCM), and the lysates were left to incubate at 4 °C overnight. The cell lysate was then clarified by centrifugation at 15,000 g at 4 °C for 15 min. The pre-cleared cell lysate was then directly incubated with pre-equilibrated Flag magnetic agarose or Strep tag II magnetic beads at 4 °C for 30 min. The proteins bound to the agarose or beads were eluted using SDS gel loading buffer and analyzed by SDS-PAGE. For endogenous Co-IP assay, cell lysate was incubated with IgG or IRG1 antibody at 4 °C for 30 min.

### Unbiased proteomics analysis

Unbiased proteomics analysis was performed at the High Throughput Screening Platform at Shanghai Institute for Advanced Immunochemical Studies at ShanghaiTech University. OFLML3-interacting proteins was isolated following a previously established protocol 27. Briefly, RAW264.7 cells were transfected with a plasmid encoding OLFML3-Myc/Flag or OLFML3-Myc/Flag/Strep. After stimulation with LPS (Cat# L2630, Sigma-Aldrich) for 12 h, the cells were collected and lysed using ice-cold IP lysis buffer (Pierce). The interaction partners of OLFML3 were extracted from the soluble fraction of the cell lysates using either Flag magnetic agarose (Cat# A36797, Pierce) or Strep magnetic beads (Cat# 2-4090, IBA Lifesciences, Göttingen, Germany) through immunoprecipitation. The resultant purified protein complexes were separated via SDS-PAGE and subjected to analysis using liquid chromatography (LC)-mass spectrometry (MS).

For analysis of the results, protein sequences were searched in the protein database. Baseline signals or hits in the control cell group without OLFML3 overexpression were subtracted from the test group. The raw data can be found in the Supplementary Source Data file.

### Measurement of macrophage itaconate and succinate

After LPS stimulation for 6 h, cells were harvested and processed by Metware Biotechnology (Wuhan, China) and OE Biotechnology (Shanghai, China). The reagents and solvents used for this experiment were either analytical or HPLC grade. Water, methanol, acetonitrile, pyridine, n-hexane, and methoxylamine hydrochloride (97%) were procured from Thermo Fisher Scientific (Waltham, MA, USA). Chloroform was purchased from Titan Technology Co, Ltd. (Shanghai, China). For sample preparation, 500 μL of pre-chilled methanol-water (4:1, V/V) solution was added to cell pellets and transferred to a 1.5 mL tube. Chloroform was then added to each tube, and the samples were dispersed by pipetting and then ultrasonic homogenization at 500 W for 20 min on ice. The raw mixture was then centrifuged at 13,000 rpm at 4 °C for 10 min. The supernatant was transferred to a glass vial and dried using a freeze concentration centrifugal dryer.

The dried materials were dissolved in a solution of 15 mg/mL methoxylamine hydrochloride in pyridine. The solution was vigorously vortexed for 2 min and incubated at 37 °C for 90 min. N,O-Bis (trimethylsilyl) trifluoroacetamide (BSTFA) (with 1% chlorotrimethylsilane (TMCS)) and n-hexane were then added, and the mixture was vigorously vortexed for 2 min and then kept at 70 °C for 60 min. Prior to GC-MS analysis, the samples were allowed to equilibrate at room temperature for 30 min. Equilibrated samples were analyzed using a Trace1310 gas chromatography coupled to a TSQ9000 MS with an electron impact (EI) source (Thermo Fisher). A DB-5MS fused-silica capillary column (30 m × 0.25 mm × 0.25 μm, Agilent J & W Scientific, Folsom, CA, USA) was employed for separation of the derivatives. Helium (>99.999%) was used as the carrier gas at a constant flow rate of 1.2 mL/min through the column. The injector temperature was maintained at 280 °C, and the injection volume was 1 μL in splitless mode. The initial oven temperature was set to 50 °C and held for 0.5 min, followed by a ramp to 125 °C at a rate of 15 °C/min, holding for 2 min, then ramping to 210 °C at a rate of 4 °C/min and holding for 3 min. Finally, the temperature was held at 305 °C for 3 min. The temperatures of the MS quadrupole and ion source (electron impact) were set to 280 °C and 300 °C, respectively. MS data was acquired in full-scan mode (m/z 40-600), with a solvent delay time set to 4 min. Quality control samples (QCs) were injected at regular intervals during the analytical run to assess repeatability.

### Phagocytosis assay

Cayman's Phagocytosis Assay Kit (IgG FITC, Cat# 500290, Cayman, Ann Arbor, MI, USA) was used to measure the phagocytic process of macrophages *in vitro*, where latex beads coated with FITC-labeled rabbit IgG was the phagocytosis probe. Cells were seeded at a density of 2×10^5^/mL culture. At 24 h after seeding, the latex beads-rabbit IgG-FITC complex was diluted at a ratio of 1:200 with pre-warmed DMEM and then used to treat the attached cells. After incubation for 2 h, DMEM was removed and Trypan Blue solution was added to 1× dilution to remove the surface-bound beads. Then cells were gently cleaned with washing solution twice. For fluorescence microscopy, cells were incubated at 37 °C for 10 min with Hoechst 33342. For flow cytometry, cells were cleaned twice and then re-suspended with washing solution immediately for flow cytometry analysis.

### Transwell assay

Cells were resuspended with serum-free medium and cell density was adjusted to 2×10^5^/well to ensure a moderate number of migrating cells, and 200 μL cell suspension was inoculated on the upper layer of the Transwell plate with 700 μL medium containing 10% FBS (with or without 1 μg/mL LPS) on the lower layer. After culture at 37 °C for 24 h, the medium below Transwell plate was discarded, gently washed once with PBS, and the cells were fixed with 4% paraformaldehyde at room temperature for 30 min. Then, the Transwell chamber was removed by fixed staining with crystal violet solution for 5-10 min. The remaining crystal violet solution and adherent cells in the upper layer of the chamber were gently removed with a cotton swab, and the chamber components were dried at 37 °C for 12-24 h. After drying, the migrating cells in the lower layer of the Transwell chamber were observed and counted under the microscope and photographed for counting and statistical analysis.

### ATP measurement

RAW264.7 cells were treated with 100 ng/mL LPS and cells were harvested at 6, 12 or 24 h after stimulation. Collected cells were lysed on ice and the ATP content was determined using an enhanced ATP assay kit (Cat# S0027, Beyotime) following the manufacturer's protocol. Luminescence signals (counts per second, CPS) were measured with the Envision multi-mode plate reader (PerkinElmer, Waltham, MA, USA).

### Immunofluorescence and confocal microscopy

Cells were seeded on to glass-bottom dishes (Nunc, Roskilde, Denmark). For mitochondrial staining, cells were pretreated with Mitotracker (Cat# M22426, Invitrogen) for 30 min prior to fixation. For fixation, cells were treated with 4% paraformaldehyde at room temperature for 15 min. To mitigate non-specific binding, cells were incubated in an immunofluorescence blocking buffer (CST) for 1 h at room temperature. The cells were then incubated with primary antibodies against Flag (1:1000, CST) and HA (1:1000, CST) at 4 °C overnight. Following three PBS washes, cells were subjected to a 1 h incubation at room temperature with the corresponding secondary antibodies (1:1000, Thermo Fisher), and nuclear staining was achieved using Hoechst 33342 (Cat# H3570, Invitrogen). Cells were imaged using a confocal microscope (LSM710, Zeiss, Oberkochen, Germany). For staining of endogenous IRG1 (1:100, Abcam), cells were fixed and permeabilized with ice-cold 100% methanol for 15 min at -20 °C. The colocalization of IRG1 and mitochondria was determined as correlation coefficient by the Coloc2 plugin in ImageJ. Mitochondrial membrane potential (MMP) was determined using MMP assay kit (Cat# C2006, Beyotime) with 5,5′,6,6′-Tetrachloro-1,1′,3,3′-tetraethyl-imidacarbocyanine (JC-1) following supplier's instructions.

To measure the co-localization of OLFML3 and IRG1, we engineered HeLa cells to stably express OLFML3-OFP and IRG1-GFP in the absence or presence of 1 μg/mL LPS. We conducted time-lapse live-cell imaging under controlled conditions at 37 °C, maintaining both humidity and CO_2_ levels in a specially designed microscopy chamber. This chamber was attached to ZEISS LSM980 confocal microscope, outfitted with a 63X oil DIC objective. For acquiring 3-D images, we utilized ZEISS ultra-high resolution microscope Elyra 7 with Lattice SIM² system.

### Flow cytometry

BMDMs purity was assessed by flow cytometry analysis using F4/80 and CD11b antibodies (Cat# 123116, Cat# 101206, Biolegend, San Diego, CA, USA). Cells were rinsed once with ice-cold PBS, suspended in a chilled staining buffer and then incubated with the specified antibodies for 20 min, followed by two washes with ice-cold PBS. Total reactive oxygen species (ROS) and mitochondrial ROS within the macrophages were quantified by exposing cells to DCFH-DA and MitoSOX fluorescent probes respectively (Cat# 50101ES01, Cat# 40778ES50, Yeasen). The fluorescence was analyzed by flow cytometry. In each experimental condition, 10,000 cells were analysed, and all experiments were executed using a CytoFLEX flow cytometer (Beckman Coulter). For single-cell sorting, experiments were conducted utilizing the MoFlo Astrios EQ Cell Sorter from Beckman Coulter.

### Native PAGE and 2D PAGE

Native gel electrophoresis was conducted in accordance with a previously established protocol 28, with slight modifications. Cells were first lysed using ice-cold native lysis buffer (Cat# BN2008, Invitrogen) and the lysates were clarified through centrifugation at 20,000 g for 30 min at 4 °C. Following the addition of 0.25% Coomassie G250 (Cat# BN2004, Invitrogen), protein separation was carried out using a 4%-16% gradient native polyacrylamide gel electrophoresis (PAGE), and the separated proteins were subsequently subjected to western blot analysis. In the case of 2D analysis, protein separation in the first dimension was accomplished using Native PAGE (Cat# BN1002BOX, Invitrogen), and the proteins from a single lane of the gel were analyzed in the second dimension through SDS-PAGE (Cat# NP0326BOX, Invitrogen), as described previously.

### Mitochondrial fractionation

Mitochondrial fractionation and proteolysis experiments were conducted following the manufacturer's instructions for the kit (Cat# 89874, Invitrogen). Briefly, 2 × 10^7^ cells were collected in a 2.0 mL microcentrifuge tube by centrifugation at 850 g for 2 min. The supernatant was removed and the cell pellet was resuspended using 800 μL of Mitochondria Isolation Reagent A. The resuspended cells were incubated on ice precisely for 2 min and then transferred to a Dounce Tissue Grinder (Cat# D8938, Sigma-Aldrich). Cells were homogenized on ice with sufficient strokes and then 800 μL of Mitochondria Isolation Reagent C was added. This mixture was centrifuged at 700 g for 10 min at 4 °C, and the supernatant was transferred to a new 2.0 mL tube and centrifuged at 12,000 g for 15 min at 4 °C. The resulting supernatant was regarded as cytosol fraction and then transferred to a new tube. The cell pellet containing isolated mitochondria was resuspended in 500 μL of Mitochondria Isolation Reagent C, followed by centrifugation at 12,000 g for 5 min. Then the supernatant was discarded and the mitochondrial pellet was kept on ice for downstream processing.

For the proteinase K resistance assay, the isolated mitochondrial pellet was washed once with Mitochondria Isolation Reagent A. Then the pellet was resuspended in mitochondrial storage solution (Cat# C3601-3, Beyotime) and treated with proteinase K (100 ng/mL), in the absence or presence of 1% Triton X-100. The proteolytic reaction was conducted on ice for 20 min, and the reaction products were then analyzed by SDS-PAGE using specified antibodies.

### High-performance liquid chromatography (HPLC) experiments

RAW264.7 cells were treated with 100 ng/mL LPS and cells were harvested at 6 h after stimulation. Collected cells were lysed by sonication in PBS supplemented with protease inhibitors and a phosphatase inhibitor (Cat# P002, NCM Biotech, Suzhou, China) on ice and then centrifuged at 12,000 rpm (13,523 × g) at 4 °C for 10 min to remove cell debris. The collected supernatants were resolved by HPLC on an Agilent 1260 column (5 μm, 250×4.6 mm). The mobile phases consisted of PBS at a flow rate of 0.35 ml/min. The column temperature was set at 25 °C. Absorption at 280 nm were recorded throughout the run. The efflux from 4.607 min to 7.007 min were collected and analyzed by western blotting. Bovine serum albumin (BSA) of 66 kD and a CRISPR cascade (CRISPR-associated complex for antiviral defense) of 405 kD 29 were used as molecular weight markers.

### Statistical analysis

Graphs were generated with GraphPad Prism v.8.2.1. Data represented mean ± SD or SEM as indicated in each figure legend. Statistical analysis was performed using unpaired two-tailed Student's t test (for comparing two groups) or two-way analysis of variance (ANOVA) followed by Tukey's multiple comparisons test (for comparing multiple groups) by GraphPad Prism. The Gehan-Breslow-Wilcoxon test was used for the analysis of survival data. The *p* values were plotted in the figure and those less than 0.05 were denoted statistically significant.

## Results

### OLFML3 suppresses lung inflammation and injury in mice

**Figure 1:**
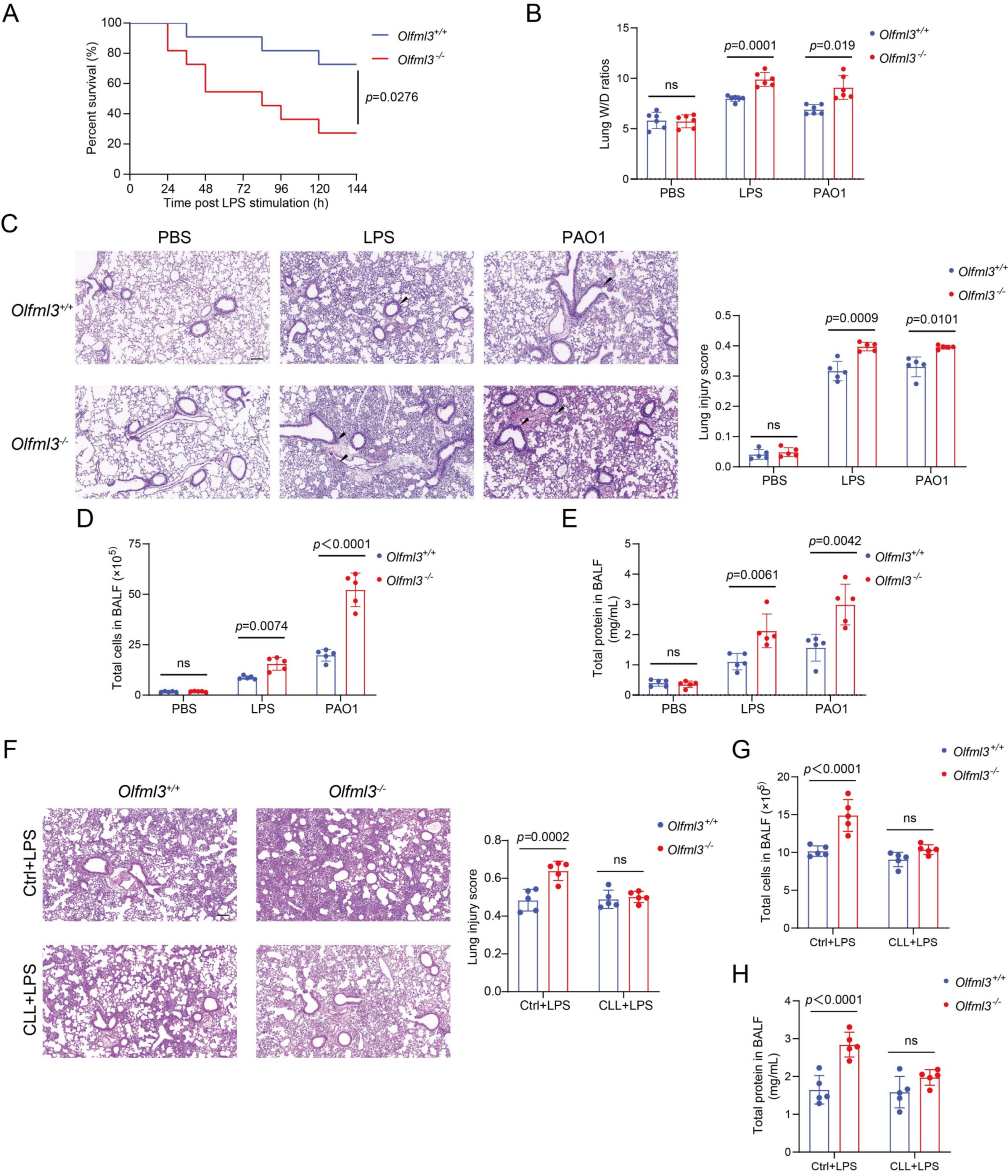
***Olfml3* knockout elevates pulmonary inflammation in LPS- or PAO1-stimulated mice.** (A) Survival curve of wild-type and *Olfml3* knockout mice challenged with 10 mg/kg LPS (*n* = 11 per group). LPS is intraperitoneally administrated to induce sepsis. (B) Evaluation of LPS- or PAO1-induced pulmonary edema, as determined by lung wet-to-dry ratio. (C) H&E staining of lung sections for analysis of inflammatory cell infiltration upon LPS and PAO1 stimulation. Scale bar, 100 μm. Black arrows indicate infiltrating cells. (D) Analysis of the total cell counts in BALF. (E) Determination of the concentrations of total proteins in BALF using BCA methods. (F) H&E staining of lung sections for analysis of alveolar macrophage depletion in *Olfml3*^+/+^ and *Olfml3*^-/-^ mice. Scale bar, 100 μm. (G, H) Analysis of the total cell counts (G) and total proteins (H) in BALF from alveolar macrophage-depleted *Olfml3*^-/-^ mice. For B to H, LPS (10 mg/kg) or PAO1 (2×10^6^ colony-forming units, CFU) is intranasally instilled to mice (*n* = 5 or 6 per group). The data are presented as mean ± SEM of three independent experiments.

We first investigated the *in vivo* functions of OLFML3 in LPS- or *Pseudomonas aeruginosa* (PAO1, ATCC-BAA-47; strain HER-1018)-induced acute lung injury (ALI) model in mice using a global *Olfml3* knockout mouse line derived from C57BL/6 background. It was found that *Olfml3* knockout significantly reduced the survival of mice during LPS-induced sepsis (Figure 1A). In addition, analysis of the lung wet/dry (W/D) ratios showed that *Olfml3* knockout exacerbated pulmonary edema in mice (Figure 1B). Under LPS or PAO1 challenge, *Olfml3^-/-^* mice displayed significantly elevated inflammatory cell infiltration surrounding airway lumens and vessels (Figure 1C) and increased total cell counts and total protein levels in bronchoalveolar lavage fluid (BALF) compared to wild-type (*Olfml3^+/+^*) mice (Figure 1D, 1E). Consistently, several pro-inflammatory cytokines were also found to be elevated in the BALF and sera of *Olfml3^-/-^* mice (Supplemental Figure 1A, 1B). These results suggested that OLFML3 played a regulatory function in LPS- and PAO1-induced ALI murine model.

Next we sought to explore the cell types that were involved in OLMLF3-mediated inhibition of inflammation in mice. It has been reported that olfactomedin 4 (OLFM4), which possesses an OLF domain with considerable similarity to that of OLFML3 6, 30 (Supplemental Figure 1C), plays critical roles in innate immunity, inflammation, and infection 31, 32. Particularly, recent study revealed a connection between OLFM4 and macrophages in ulcerative colitis 33. To examine whether macrophages were related to the *in vivo* function of OLFML3, we performed clodronate liposomes (CLL) macrophage depletion experiments 34. CLL was intranasally instilled into wild-type and *Olfml3^-/-^* mice to deplete alveolar macrophages. At 48 h after CLL treatment, LPS was intranasally administrated to establish the sepsis model. Our results showed that the effect of OLFML3 on inflammatory cell infiltration (Figure 1F) was largely abolished in the macrophage-depleted mice. Consistent results were observed with total cells and total proteins in the BALF (Figure 1G, 1H). These results indicated that OLFML3 exerted *in vivo* modulatory function via affecting macrophage functions.

### OLFML3 promotes macrophage phagocytosis and migration

In order to understand how OLFML3 affected the functions of macrophages during response to PAMPs, we performed RNA-Seq analysis on *Olfml3* knockout RAW264.7 cells and BMDMs in the absence and presence of LPS. The biological replicates exhibited consistent gene clustering and sample correlation patterns within the same groups, as displayed by a self-organizing learning algorithm 24, 25 (Supplemental Figure 2A). A number of differentially expressed genes (DEGs) were identified between wild-type and *Olfml3* knockout cells with and without LPS treatment (Supplemental Figure 2B, 2C). Gene ontology (GO) analysis of identified DEGs in RAW264.7 and BMDMs revealed terms including response to external stimulus and cell migration (Supplemental Figure 2D, 2E).

**Figure 2:**
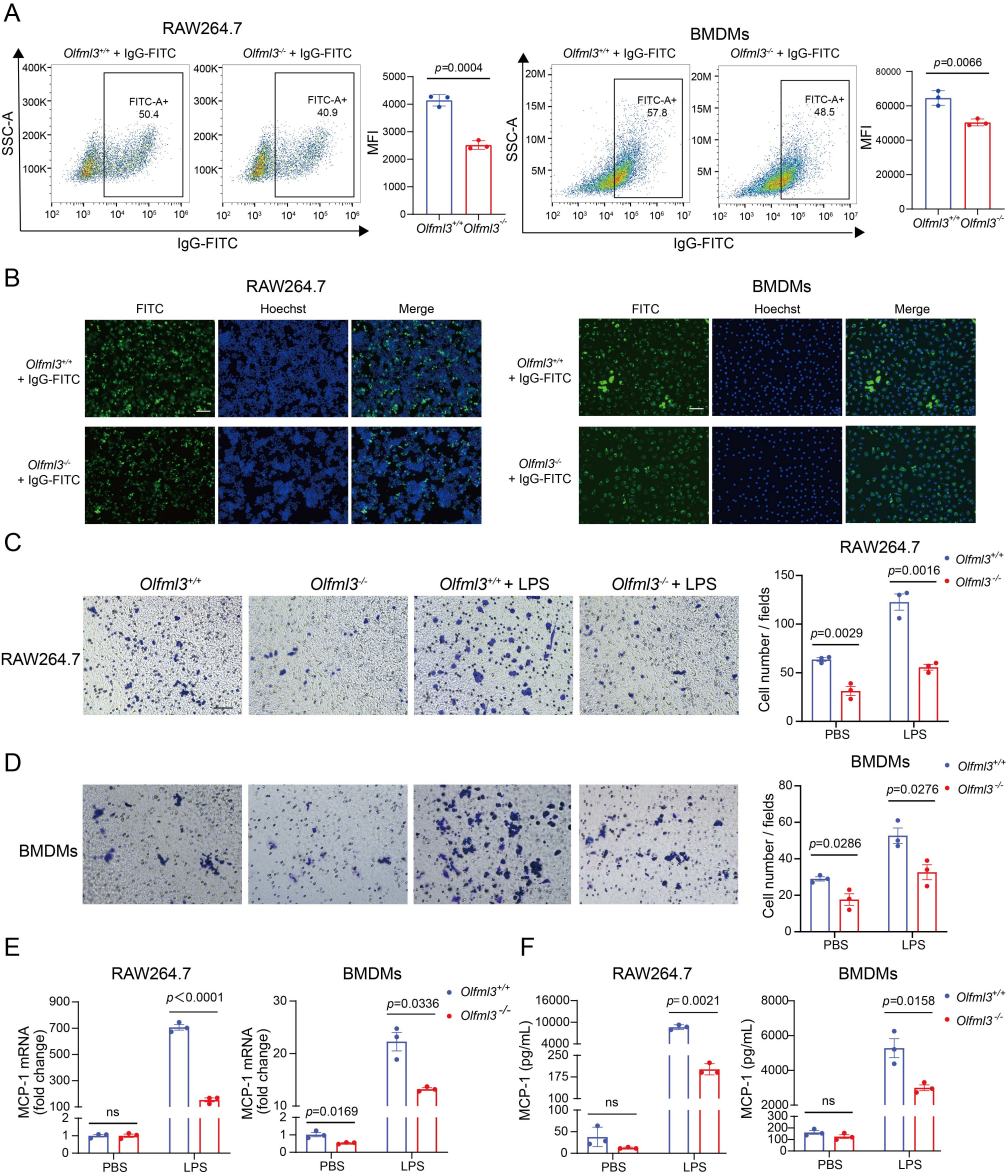
***Olfml3* knockout reduces the phagocytic and migration functions of macrophages.** (A, B) Analysis of the phagocytic function of RAW264.7 (left) and BMDMs (right) using latex beads carrying FITC-labeled IgG using flow cytometry (A) and immunofluorescence (B) experiments. The cells are incubated with the beads for 2 h. (C, D) Evaluation of the effects of OLFML3 on the migration of RAW264.7 (C) and BMDMs (D) in the absence or presence of LPS using transwell assay. At 24 h after 1 μg/mL LPS stimulation, the transmembraned cells are counted from three microscopic fields with 40× magnification. One representative microscopic field of each group is shown on the left (Magnification 20×; Scale bar, 150 μm). The bar graphs show the averaged values of three biological replicates. (E, F) Determination of MCP-1expression at intracellular mRNA (E) and soluble protein (F) levels in RAW264.7 (left) and BMDMs (right), using RT-qPCR and ELISA respectively. The *Olfml3*^-/-^ cells are isolated single clones containing gene knockout at both alleles of *Olfml3*. For E, cells are collected at 4 h post 100 ng/mL LPS stimulation. For F, culture supernatant is collected at 24 h after 100 ng/mL LPS stimulation. The data are the mean ± SEM of three biological replicates. ns, not significant.

Guided by this transcriptome-wide information, we examined the effects of *Olfml3* knockout on macrophage phagocytosis and migration. Flow cytometry (Figure 2A; Supplemental Figure 3A) and immunofluorescence (Figure 2B) experiments showed that *Olfml3^-/-^* RAW264.7 and BMDMs had significantly decreased phagocytosis of IgG-FITC-immobilized latex beads compared to *Olfml3^+/+^* macrophages. Moreover, transwell assay showed that *Olfml3* knockout dampened the migration of RAW264.7 and BMDMs both in the absence and presence of LPS (Figure 2C, 2D). Interestingly, *Olfml3* knockout in RAW264.7 cells reduced the expression of monocyte chemoattractant protein-1 (MCP-1) at mRNA and protein levels in response to LPS stimulation (Figure 2E, 2F; Supplemental Figure 3B), suggesting that OLFML3 was involved in the positive feedback of macrophage recruitment as described 35-38. Importantly, the above phenotypical changes by *Olfml3* knockout were unlikely due to indirect effects of cell growth because EdU assay revealed similar cell proliferation between *Olfml3^+/+^* and* Olfml3^-/-^* RAW264.7 cells (Supplemental Figure 3C). Collectively, the above data showed that OLFML3 could promote phagocytosis and migration of macrophages.

### Identification of IRG1 as an OLFML3-interacting protein

**Figure 3:**
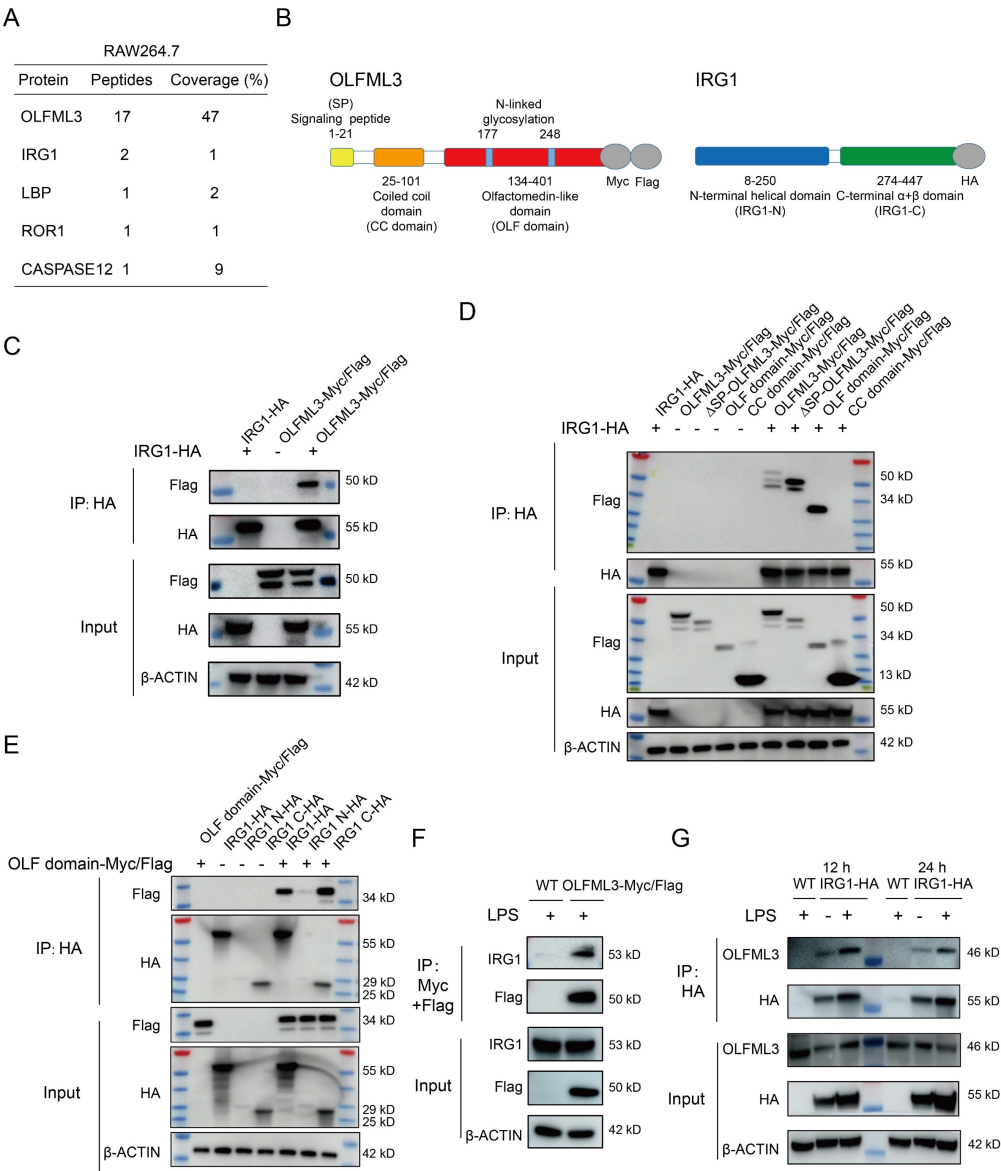
** Dissection of the pattern of interactions between OLFML3 and IRG1.** (A) Identification of IRG1 as an OLFML3-interacting protein in RAW264.7 by mass spectrometry analysis of OLFML3-Myc/Flag co-immunoprecipitated proteins. OLFML3-interacting proteins are enriched using Flag magnetic beads. The top 5 candidate proteins are shown. (B) Schematic presentation of OLFML3 and IRG1 domain organization. (C) Validation of the interactions between OLFML3 and IRG1 in HEK293T cells. OLFML3-Myc/Flag is co-expressed with IRG1-HA, immunoprecipitated (IP) by HA beads and immunoblotted (IB) with Flag antibody. (D) Investigation of IRG1-interacting OLFML3 domains in HEK293T cells. Myc/Flag-tagged OLFML3 constructs are co-expressed with IRG1-HA, immunoprecipitated by HA beads and immunoblotted with Flag antibody. (E) Investigation of OLF domain-interacting IRG1 domains in HEK293T cells. HA tagged IRG1 constructs are co-expressed with OLF-Myc/Flag, immunoprecipitated by HA beads and immunoblotted with Flag antibody. (F) Validation of the interactions between OLFML3-Myc/Flag and endogenous IRG1 in a RAW264.7 cell line stably expressing OLFML3-Myc/Flag. The cells are treated with 100 ng/mL LPS for 12 h. Cell lysate is immunoprecipitated with a sequential two-step affinity purification with Myc and Flag magnetic beads and then immunoblotted with an IRG1 antibody. (G) Validation of the interactions between IRG1-HA and endogenous OLFML3 in a RAW264.7 cell line stably expressing IRG1-HA. The cells are treated with 100 ng/mL LPS for 12 h or 24 h. Cell lysate is immunoprecipitated with an affinity purification with HA magnetic beads and then immunoblotted with an OLFML3 antibody.

To determine molecular mechanism of OLFML3 in macrophages, we performed mass spectrometry analysis on OLFML3 co-immunoprecipitated cellular proteins to seek for OLFML3 partner proteins. A mitochondria-localized protein IRG1 39 was identified as one of the OLFML3-interacting proteins in macrophages (Figure 3A). *Irg1* gene encodes an aconitate decarboxylase that catalyzes the conversion of Kreb's cycle intermediate *cis*-aconitate to itaconate 40 in macrophages and other myeloid cells. Mouse IRG1 (mIRG1) and human IRG1 (hIRG1) share 85% sequence identity 41. Structurally, IRG1 adopts the fold of MmgE/PrpD protein family, which contains a larger N-terminal all helical domain and a smaller C-terminal α+β domain 42. Interestingly, a very recent study reported that IRG1 and itaconate could promote macrophage phagocytosis during bacterial infection, which was similar to the observed activity of OLFML3 in macrophages in the present study 43.

To determine the mode of interaction between OLFML3 and IRG1, we generated several OLFML3 and IRG1 truncation constructs for co-immunoprecipitation (Co-IP) analyses. For OLFML3, the constructs included full-length protein (OLFML3), protein deficient of signal peptide (ΔSP-OLFML3), coiled-coil domain (CC domain) and olfactomedin domain (OLF domain), all of which contained dual C-terminal Myc and Flag tags (Figure 3B). For IRG1, the constructs included full-length protein (IRG1), N-terminal helical domain (IRG1-N) and C-terminal α+β domain (IRG1-C), all bearing a C-terminal HA tag (Figure 3B).

Ectopic expression of OLFML3 constructs and IRG1 constructs were performed in HEK293T cells. We first confirmed the MS results of the interactions between full-length OLFML3 and full-length IRG1 under physiological condition (Figure 3C). Further analyses showed that IRG1 could be co-immunoprecipitated with full-length OLFML3, ΔSP-OLFML3 and OLF domain, but not with CC domain (Figure 3D). These results suggested that OLFML3 interacted with IRG1 via its OLF domain. Next we assessed which portion of IRG1 interacted with the OLF domain of OLFML3. It was found that OLF domain could effectively interact with the full-length IRG1 and IRG1-C, with trace signal detected for IRG1-N (Figure 3E). Given the poor expression of IRG1-N (Figure 3E, HA input), the possibility of interactions between OLF and IRG1-N could not be excluded.

Furthermore, we intended to validate the pattern of interactions between OLFML3 and IRG1 as endogenous proteins. We first established stable RAW264.7 cell lines expressing OLFML3-Myc/Flag. Following LPS stimulation, a two-step affinity purification using Myc and Flag magnetic beads was performed and endogenous IRG1 was detected as an OLFML3-Myc/Flag-interacting protein by Flag immunoprecipitation (Figure 3F). In the same rationale, we established stable RAW264.7 cell lines expressing IRG1-HA and found that endogenous OLFML3 could interact with IRG1-HA at 12 h or 24 h post LPS stimulation (Figure 3G).

To gain more insights on the interactions between OLFML3 and IRG1 under physiological conditions, we employed native PAGE and two-dimensional (2D) SDS-PAGE to examine the formation of intracellular complex in response to LPS. We constructed RAW264.7 cells stably expressing OLFML3-Myc/Flag, ΔSP-OLFML3-Myc/Flag or CC domain-Myc/Flag constructs and analyzed the intracellular complex of endogenous IRG1 and the corresponding OLFML3 constructs. On 1D native PAGE, higher molecular weight bands were identified with full-length OLFML3 and ΔSP-OLFML3 (Supplemental Figure 4A, 4B), but not with CC domain, (Supplemental Figure 4C). This result was consistent with the Co-IP analyses for the pattern of interactions between OLFML3 and IRG1 (Figure 3). Resolving the higher molecular weight bands from 1D native PAGE on 2D denatured SDS-PAGE uncovered overlapped OLFML3-Myc/Flag and IRG1 (Supplemental Figure 4A, 4B), suggesting the formation of OLFML3-IRG1 complex. In addition, we found that *Olfml3* knockout reduced IRG1 protein level in RAW264.7 and BMDMs at basal (zero time point) and early (before 12 h) stage upon LPS stimulation (Supplemental Figure 4D, 4E).

### OLFML3 prevents mitochondrial membrane potential (MMP) loss and mitochondrial reactive oxygen species (mtROS) over-production in LPS-stimulated macrophages

Following the confirmation of OLFML3-IRG1 interaction, we next asked the physiological meaning of this interaction and how it determined the effects of OLFML3 on macrophage function. We re-visited the RNA-Seq data and found that upon LPS stimulation, the biological processes related to oxidative stress were enriched in *Olfml3* knockout RAW264.7 and BMDMs (Supplemental Figure 2). This finding indicated that OLFML3 had inhibitory effects on LPS-induced oxidative stress, which aligned with the function of IRG1/itaconate on inhibiting LPS-induced inflammation and oxidative stress 44-48.

**Figure 4:**
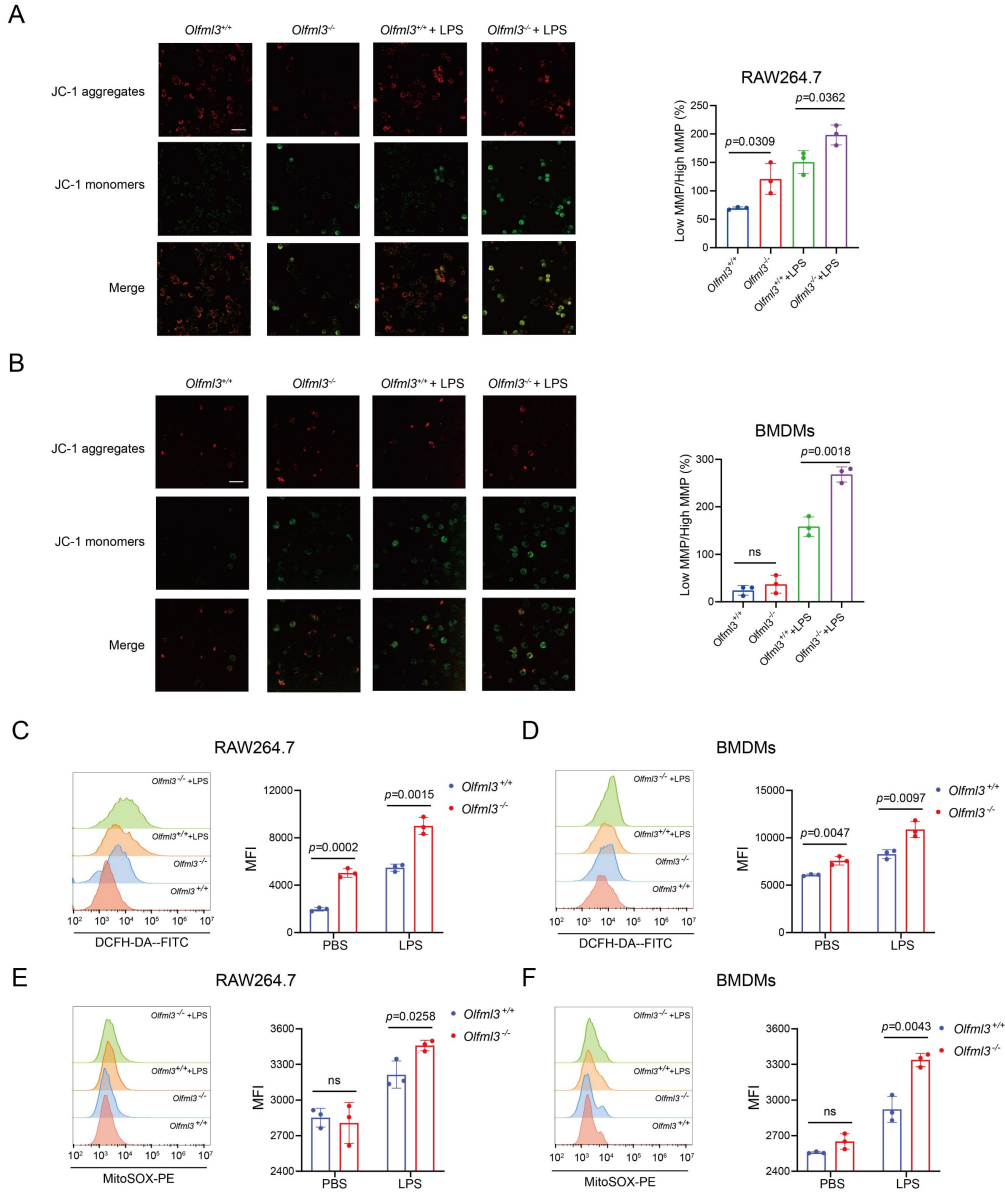
***Olfml3* knockout alters MMP and mitochondrial ROS production in macrophages.** (A, B) Evaluation of the effects of OLFML3 on LPS-stimulated MMP changes in RAW264.7 (A) and BMDMs (B) using JC-1 fluorescence probe. (C, D) Analysis of the effects of OLFML3 on total cellular ROS in RAW264.7 (C) and BMDMs (D) using DCFH-DA fluorescence probe. (E, F) Analysis of the effects of OLFML3 on mtROS in RAW264.7 (E) and BMDMs (F) in response to LPS treatment using MitoSOX fluorescence probe. Cells are collected at 12 h post 100 ng/mL LPS stimulation. MFI, median fluorescent intensity. The data represent mean ± SEM of at least three biological replicates. ns, not significant.

As IRG1 was localized on mitochondria, we thus hypothesized that OLFML3 exerted its antioxidant activity by affecting the functions of mitochondria. We first employed JC-1 probe to characterize mitochondrial membrane potential (MMP, ∆Ψm) in *Olfml3* knockout macrophages, where the probe aggregated at high MMP to show red fluorescence and became monomer at low MMP to give green fluorescence 49-51. It was found that upon LPS stimulation, *Olfml3* knockout consistently reduced MMP in RAW264.7 and BMDMs, as evidenced by the higher ratio of low MMP over high MMP (Figure 4A, 4B).

Moreover, DCFH-DA fluorescent probe analysis showed that *Olfml3^-/-^* RAW264.7 and BMDMs displayed significantly higher total cellular ROS than *Olfml3^+/+^* cells in the presence of LPS (Figure 4C, 4D; Supplemental Figure 5A, 5B). More importantly, *Olfml3* depletion increased LPS-induced mitochondrial ROS (mtROS) production (Figure 4E, 4F; Supplemental Figure 5C, 5D). In addition, Mito-TEMPO, a commonly used mtROS scavenger, could revert the effects of *Olfml3* knockout on LPS-induced mtROS production (Supplemental Figure 5E).

Collectively, these results showed that the presence of OLFML3 could prevent LPS-induced MMP loss and mtROS over-production in macrophages. Previous studies have reported that mitochondrial dysfunction including MMP loss and mtROS over-production is associated with macrophage phagocytosis and migration 52-55. The *Olfml3* loss-of-function studies on macrophages (Figure 2) and on mitochondria (Figure 4) indicated that OLFML3 promoted macrophage phagocytosis and migration by preventing mitochondrial dysfunction.

### The effects of OLFML3 on mitochondrial functions are associated with its activity on IRG1-mediated metabolic processes

**Figure 5:**
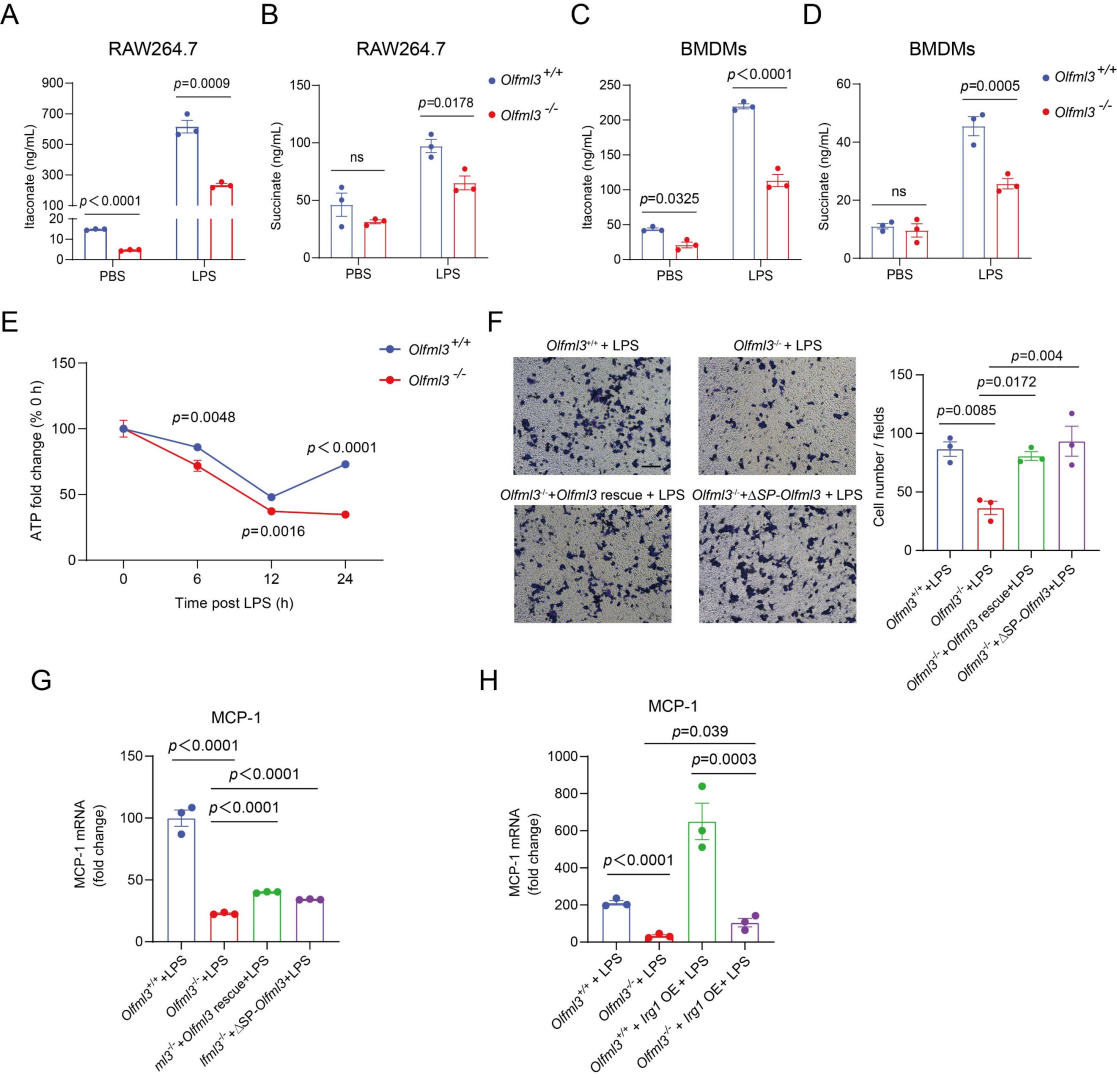
**OLFML3 affects macrophage functions by involving in IRG1-mediated metabolic processes.** (A, B) Intracellular itaconate (A) and succinate (B) levels in wild-type and *Olfml3^-/-^* RAW264.7 cells treated with LPS for 6 h. (C, D) Intracellular itaconate (C) and succinate (D) levels in wild-type and *Olfml3^-/-^* BMDMs treated with LPS for 6 h. (E) Changes of ATP levels in LPS-treated wild-type and *Olfml3^-/-^* RAW264.7 cells over a course of 24 h. (F, G) *Olfml3* overexpression rescue of damaged migration ability in *Olfml3^-/-^* RAW264.7 cells, as evaluated by transwell assay (F) and MCP-1 expression (G). For F, transmembraned cells are counted from three microscopic fields (with 40× magnification) in each membrane (three independent biological replicates per group) at 24 h after treatment with 1 μg/mL LPS. One representative microscopic field of each group is shown (magnification, 20×; Scale bar, 150 μm). For G, MCP-1 mRNA expression is determined by RT-qPCR at 4 h after treatment with 100 ng/mL LPS. (H) *Irg1* overexpression rescue of damaged migration ability in *Olfml3^-/-^* RAW264.7 cells, as evaluated by MCP-1 expression. The mRNA expression of MCP-1 is determined by RT-qPCR at 4 h after treatment with 100 ng/mL LPS. Data represent the mean ± SEM of at least three biological replicates. ns, not significant.

Next we intended to explore how OLFML3 was involved in mitochondrial functions. Based on the evidence of OLFML3-IRG1 interaction (Figure 3) and the well-established role of IRG1 in the mitochondrial metabolic processes in macrophages, we hypothesized that OLFML3 was involved in mitochondrial function via IRG1-mediated metabolic processes. We thus set a series of loss-of-function experiments to investigate the effects of *Olfml3* knockout on the metabolic processes of IRG1. Importantly,* Olfml3^-/-^* RAW264.7 exhibited significantly decreased itaconate production upon LPS stimulation, in comparison to wild-type cells (Figure 5A). Consistently, *Olfml3* knockout reduced LPS-induced production of succinate, a Kreb's cycle intermediate (Figure 5B). Similarly, *Olfml3* knockout reduced itaconate and succinate production in LPS-treated BMDMs (Figure 5C, 5D). Furthermore, compared to wild-type cells, *Olfml3^-/-^* RAW264.7 exhibited significantly lower ATP content under LPS challenge (Figure 5E), which could be the results of perturbed Kreb's cycle.

We then performed gain-of-function experiments to investigate whether the reduced migration ability of *Olfml3^-/-^* RAW264.7 could be rescued. Transwell experiments showed that overexpression of full-length OLFML3 or ΔSP-OLFML3 could rescue the migration ability of *Olfml3^-/-^* cells to the wild-type level (Figure 5F). Similar results were observed with MCP-1 expression in rescued *Olfml3^-/-^* RAW264.7 (Figure 5G).

More importantly, *Irg1* overexpression could partially rescue MCP-1 expression in LPS-treated *Olfml3^-/-^* RAW264.7 cells (Figure 5H). This result suggested that IRG1 was unlikely to be the upstream node protein of OLFML3, the knockout of which would otherwise completely block the effects of *Irg1* overexpression rescue. In addition, it has been well known that IRG1 is an aconitate decarboxylase and catalyzes the conversion of *cis*-aconitate to itaconate in mitochondria. Therefore, these data suggested that OLFML3 affected mitochondrial function via IRG1-mediated metabolic processes in mitochondria.

### Co-localization of OLFML3 and IRG1 on mitochondria

**Figure 6:**
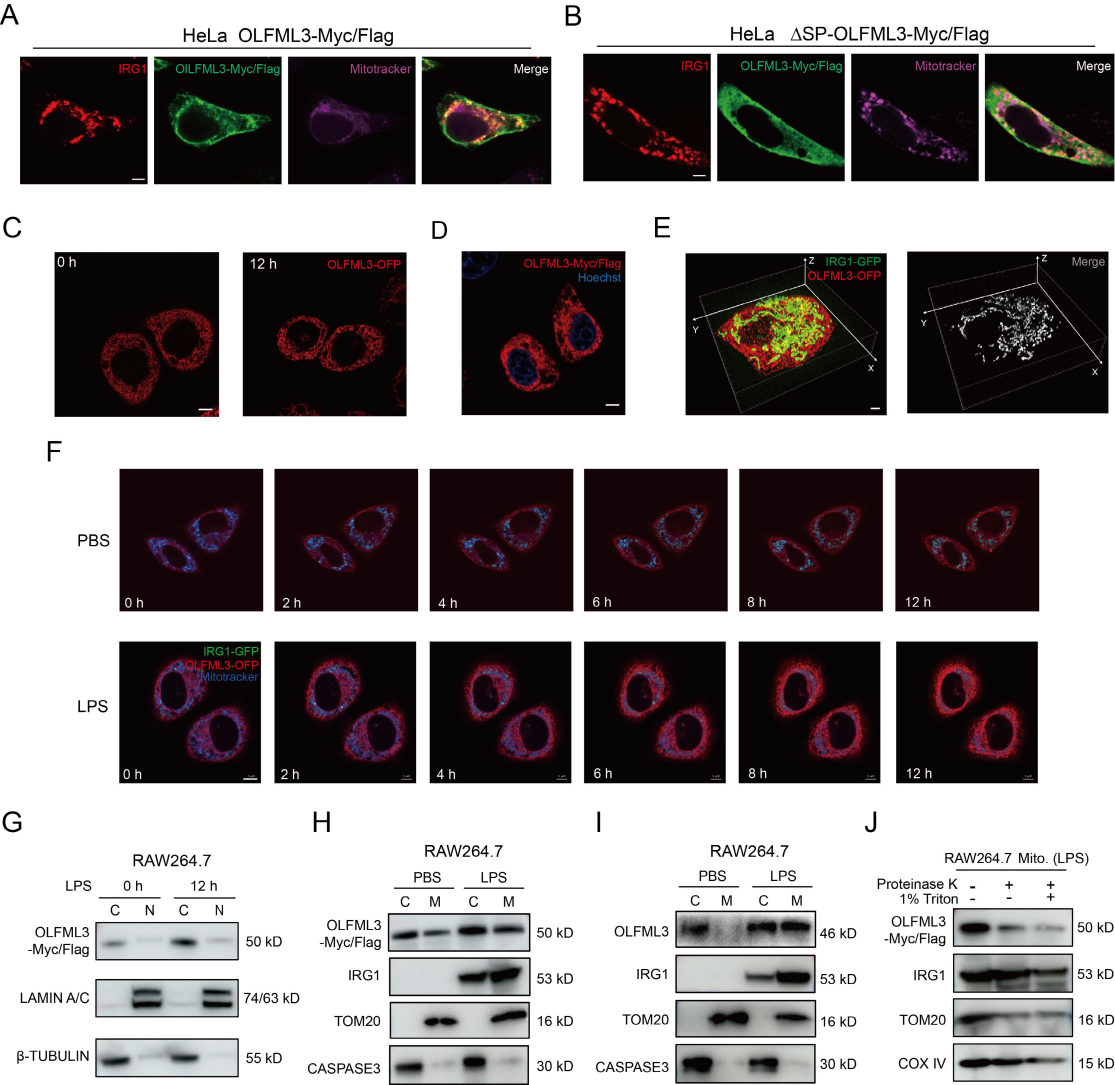
**Examination of the formation of OLFML3-IRG1 complex and the subcellular localization of OLFML3 and IRG1 proteins.** (A, B) Immunofluorescence analysis of the mitochondrial co-localization of IRG1-HA with OLFML3-Myc/Flag (A) or ΔSP-OLFML3-Myc/Flag (B) in HeLa cells. Mitochondria are stained with 200 nM Mitotracker Deep Red FM. (C) Fluorescence imaging analysis of the subcellular localization of OLFML3-OFP fusion protein in HeLa cells, treated with 1 μg/mL LPS. (D) Immunofluorescence analysis of the subcellular localization OLFML3-Myc/Flag in HeLa cells. Cells are stained with Flag antibody and Hoechst 33342. (E) Snapshot image showing 3D-SIM acquisitions of the fluorescence in LPS-stimulated HeLa cells stably expressing OLFML3-OFP (red) and IRG1-GFP (green). (F) Live-cell fluorescence time-lapse microscopy of cultured HeLa cells stably expressing OLFML3-OFP (red), IRG1-GFP (green) and Mitotracker (blue), monitored over a course of 12 h with 1 μg/mL LPS stimulation. PBS treatment is used as a control for basal changes of co-localization over time. (G) WB analysis of the cytoplasmic and nuclear contents of OLFML3- Myc/Flag in RAW264.7 treated with 100 ng/mL LPS for indicated durations. C, cytoplasmic. N, nuclear. Lamin A/C, nuclear marker. β-tubulin, cytoplasm marker. (H, I) WB analysis of the cytoplasmic and mitochondrial fractions of OLFML3 and IRG1 in RAW264.7 cell line stably expressing OLFML3-Myc/Flag (H) and wild-type RAW264.7 cells (I). Cells are treated with 100 ng/mL LPS or PBS for 12 h. The mitochondrial and cytosolic fractions of cell lysates are immunoblotted with IRG1 antibody and Flag antibody (H) or OLFML3 antibody (I) respectively. C, cytoplasmic. M, mitochondrial. Tom20, mitochondrial marker. Caspase 3, cytoplasm marker. (J) Evaluation of the sub-mitochondrial localization of OLFML3 in RAW264.7 cells stably expressing OLFML3-Myc/Flag. At 12 h after treatment with 100 ng/mL LPS, isolated mitochondrial fraction is treated with 100 ng/mL proteinase K in the absence or presence of 1% TritonX-100 for 20 min on ice and immunoblotted with antibodies as indicated.

While the above results illustrated the interaction between OLFML3 and IRG1 and involvement of OLFML3 in IRG1-mediated metabolic processes, one key question to ask was whether OLFML3 could co-localize with IRG1 on to mitochondria. Therefore, we sought to examine the subcellular localization of OLFML3 and IRG1 using imaging experiments. HeLa cells were co-transfected with plasmids encoding IRG1-HA and full-length OLFML3-Myc/Flag or ΔSP-OLFML3-Myc/Flag. It was found that IRG1 and OLFML3 were co-localized in cytoplasm and mitochondria (Figure 6A, 6B).

Moreover, we generated fluorescent protein (FP)-fused OLFML3 and IRG1 constructs to explore the dynamics of OLFML3 and IRG1 interactions. In order to exclude the effects of FP fusion on the subcellular localization of OLFML3, we constructed stable HeLa cell line expressing OLFML3 with and without orange fluorescent protein (OFP) fusion. It was found that OFP fusion did not alter the pattern of subcellular localization of OLFML3 (Figure 6C, 6D). On this basis, we constructed stable HeLa cell line co-expressing OLFML3-OFP and IRG1-GFP. Two-color three-dimensional super-resolution structured-illumination microscopy revealed intimate interactions between OLFML3 and IRG1 following LPS treatment (Figure 6E; Supplemental Movie 1). Live cell fluorescence imaging provided further evidence for LPS-induced OLFML3-IRG1 co-localization over a time course of 12 h (Figure 6F; Supplemental Movie 2, 3).

In addition to fluorescence imaging analyses, we also used cellular component fractionation to examine the subcellular localization of OLFML3 and IRG1. Analysis of RAW264.7 stably expressing OLFML3-Myc/Flag showed that intracellular OLFML3 predominantly resided in the cytoplasmic fraction rather than nuclear fraction (Figure 6G) regardless of LPS stimulation, which was consistent with the fluorescence microscopy experiments (Figure 6C). More importantly, LPS stimulation promoted the mitochondrial localization of OLFML3-Myc/Flag and IRG1 (endogenous) (Figure 6H) in RAW264.7 cell line stably expressing OLFML3-Myc/Flag. Consistently, in wild-type RAW264.7 cells, LPS treatment also increased mitochondria-localized OLFML3 (endogenous) and IRG1 (endogenous) (Figure 6I).

Next we aimed to determine the sub-mitochondrial localization of OLFML3. We performed a proteinase K membrane protection experiment to distinguish OLFML3 localization on the outer and inner membranes of mitochondria. It was found that proteinase K treatment removed the majority of mitochondria-localized OLFML3-Myc/Flag while having minor impact on mitochondrial IRG1 (Figure 6J), which was known to be present in the mitochondrial matrix 56. We thus concluded that OLFML3-Myc/Flag was mainly localized on the outer membrane of mitochondria.

### OLFML3 facilitates IRG1 mitochondrial localization via mitochondrial transport protein AIFM1

**Figure 7:**
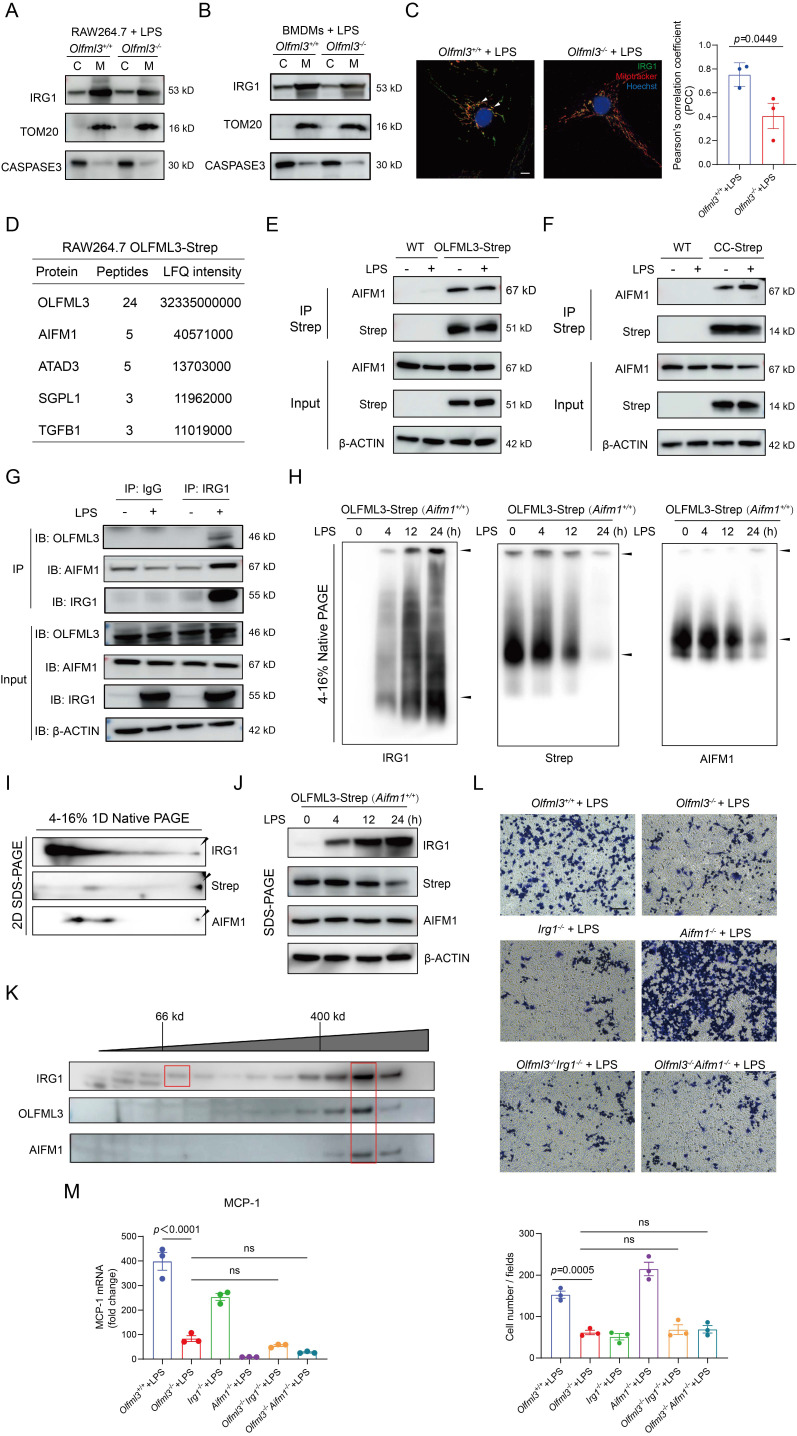
** OLFML3 facilitates IRG1 mitochondrial localization via AIFM1.** (A, B) Examination of the effects of *Olfml3* knockout on the level of mitochondrial IRG1 protein in RAW264.7 cells (A) and BMDMs (B). Cells are stimulated with 100 ng/mL LPS for 12 h and then fractionated. (C) Confocal immunofluorescence images showing the subcellular localization of IRG1-HA in wild type and *Olfml3^-/-^* BMDMs following 100 ng/mL LPS stimulation for 12 h. The bar plot shows the quantification of co-localized IRG1 (green) and mitochondria (red). The fluorescence images in this figure represent one of the three independent replicates. Scale bars, 5 μm. (D) Identification of AIFM1 as an OLFML3 interacting protein in RAW264.7 by CoIP-MS. The top 5 candidate proteins are shown. (E, F) Analysis of the interactions between AIFM1 with OLFML3-Strep (E) or CC domain-Strep (F) in RAW264.7 stable cells line. The cells stably expressing OLFML3-Strep and CC domain-Strep are treated with PBS or 100 ng/mL LPS for 12 h. The cell lysate is immunoprecipitated with Strep tag II magnetic beads and then immunoblotted with antibodies as indicated. (G) Confirmation of the interaction between OLFML3, IRG1 and AIFM1 with endogenous co-IP assay. (H) Evaluation of the presence of AIFM1 in OLFML3-IRG1 complex by native PAGE in RAW264.7 cells stably expressing OLFML3-Strep. (I) Investigation of the presence of AIFM1 in OLFML3-IRG1 complex by 2D SDS-PAGE. (J) Confirmation of the expression of IRG1, OLFML3-Strep and AIMF1 in RAW264.7 cells stably expressing OLFML3-Strep. (K) Evaluation of the presence and the molecular weight of OLFML3-IRG1-AIFM1 complex using size exclusion chromatography. The anticipated molecular weight of OLFML3-Strep, IRG1 and AIFM1 are 51, 53 and 67 kD respectively. (L, M) Comparison of the effects of *Olfml3^-/-^Irg1^-/-^* and *Olfml3^-/-^Aifm1^-/-^* double knockout with *Olfml3^-/-^* single knockout in LPS-treated RAW264.7 cells, as characterized by transwell assay (L) and MCP-1 mRNA level (M).

Because the above results revealed LPS-induced mitochondrial localization of OLFML3 and IRG1 (Figure 6F, 6H, 6I), we were curious to know whether these two partner proteins coordinated or independently proceed for mitochondrial localization. We compared the quantities of mitochondrial IRG1 in *Olfml3^-/-^* and *Olfml3^+/+^* macrophages and found that *Olfml3* knockout reduced mitochondrial IRG1 (Figure 7A, 7B). Consistent with the fractionation results, immunofluorescence analysis showed that *Olfml3* knockout in BMDMs reduced mitochondria-localized IRG1 (Figure 7C).

Because OLFML3 itself does not contain mitochondria-localizing signal, we speculated that a third partner protein might involve in OLFML3-IRG1 complex for mitochondrial localization. Inspired by a previous study where *Xenopus* OLFML3 was reported to function as a scaffold protein to interact with two partner proteins via OLF domain and CC domain respectively 5, we sought to explore whether mammalian OLFML3 could resemble *Xenopus* OLFML3 by interacting with IRG1 and a third mitochondria-localizing protein via OLF domain and CC domain respectively.

We established an additional stable RAW264.7 cell line expressing Strep tag II-fused OLFML3 (OLFML3-Strep), in order to avoid the noise associated with OLFML3-Myc/Flag. MS analysis on Strep tag II-enriched proteins identified apoptosis inducing factor mitochondria associated 1 (AIFM1) as one of the OLFML3-interacting proteins (Figure 7D). AIFM1 was known to involve in the transport of proteins from cytoplasm to mitochondria 57, 58. Co-IP analysis showed that both the full-length form and the CC domain of OLFML3 could interact with AIFM1 (Figure 7E, 7F). Furthermore, we confirmed the interaction between OLFML3, IRG1 and AIFM1 with endogenous co-IP assay (Figure 7G).

Next we intended to investigate whether AIFM1 facilitated the formation of OLFML3-IRG1 complex. Native 1D PAGE showed that higher molecular weight AIFM1 was present at the same position with the higher molecular weight components of OLFML3 and IRG1 (Figure 7H), suggesting the formation of a ternary complex. This result was further corroborated by 2D SDS-PAGE, where AIFM1, OLFML3 and IRG1 were blotted at the same position (Figure 7I, 7J). To further confirm the presence of OLFML3-IRG1-AIFM1 complex under near-physiological conditions, we conducted size exclusion chromatography (SEC) analysis of the presence of the complex in RAW264.7 cell lysate. It was found that in the cell lysate of LPS-treated RAW264.7 cells, several elution fractions above 400 kD contained all of the three proteins OLFML3, IRG1 and AIFM1 (Supplemental Figure 6A and Figure 7K), suggesting the formation of OLFML3-IRG1-AIFM1 complex.

To explore the effects of AIFM1 on the formation of OLFML3-IRG1 complex, we conducted *Aifm1* knockout in the context of stable RAW264.7 cell line expressing OLFML3-Strep (Supplemental Figure 6B, 6C). Native PAGE analyses showed that in the absence of AIFM1, OLFML3 could no longer form any complex with IRG1 (Supplemental Figure 6D, 6E). These results collectively suggested that the interaction between OLFML3 and IRG1 was dependent on AIFM1. To further investigate whether OLFML3, IRG1 and AIFM1 were in the same signaling pathway, we constructed double knockout RAW264.7 cells on top of *Olfml3* knockout (Supplemental Figure 6F) and compared the *Olfml3^-/-^Irg1^-/-^* and *Olfml3^-/-^Aifm1^-/-^* double knockout cells with *Olfml3^-/-^* single knockout cells for their migration and MCP-1 levels upon LPS treatment. The results showed that double knockout did not exacerbate the phenotypes of *Olfml3^-/-^* single knockout (Figure 7L, 7M). These results suggested that OLFML3, IRG1 and AIFM1 belong to the same signaling pathway in macrophages in response to LPS stimulation.

## Discussion

Following our previous discovery of the immunosuppressive function of OLFML3 in rhinovirus infection in HeLa-H1 cells 10, the present study has provided evidence for a broader function of OLFML3 in macrophages in response to PAMPs stimulation. While OLFML3 promoted viral infection by antagonizing type I IFN signaling, it could reduce the severity of LPS- and PAO1-induced ALI in mice by promoting macrophage phagocytosis and migration. These observations consistently reflect the immunomodulatory function of OLFML3 in viral and bacterial infection, across non-immune cells and immune cells. We showed that the function of OLFML3 on mitochondria in LPS-induced macrophages was achieved by affecting IRG1 mitochondrial localization and itaconate-related metabolic processes.

Previous studies revealed the function of OLFML3 in promoting the pro-tumorigenic function of microglia by acting as a target for tumor growth factor-β (TGF-β) 59. Unlike previous reports, the current study investigated a more general role of OLFML3 in macrophages during bacterial infection. One interesting discovery was the intracellular function of OLFML3. Due to the presence of a secretion signal peptide in protein sequence, most previous studies focused on the extracellular function of OLFML3 such as recruitment of immunosuppressive microglia 7 or tissue repair 60. Similar to the function of OLFM4 in inhibiting cathepsin C-mediated protease activities intracellularly 61, the present study demonstrated that OLFML3 could function as an intracellular regulatory protein via the interactions with a mitochondrial protein IRG1. Experiments with OLFML3 truncation constructs demonstrated that the deletion of signal peptide did not affect the subcellular localization of OLFML3 (Figure 6A, 6B) nor its interactions with IRG1 (Figure 3D). Specifically, we showed that OLFML3 was localized on the outer membrane of mitochondria (Figure 6H-6J). This observation of the intracellular function of OLFML3 along with the previous reports of its extracellular function raises an interesting question on how the dynamics of these two forms of OLFML3 is regulated under physiological and pathological conditions.

Related with the above question is a largely unaddressed point in the present study of the dynamic expression pattern of OLFML3 in macrophages or other cell types. In previous studies, OLFML3 was mostly recognized as a microglia-specific protein that has little or no expression in immune cells 7, 8, 12-14. Nevertheless, it was found in our recent study that OLFML3 mRNA expression in HeLa cells was upregulated for more than 400 folds upon RV infection 10. In the present study, it seemed that LPS could increase the total cellular protein of OLFML3 (Figure 6I, the total of cytoplasmic and mitochondrial fractions). These data suggested that the expression of OLFML3 was under precise regulation that could respond to external stimuli. In addition, fluorescence imaging and fractionation studies consistently showed that LPS stimulation on macrophages could trigger the mitochondrial localization of OLFML3 (Figure 6F, 6H, 6I). Further studies are thus required to illustrate the molecular basis and biological implications of the regulation of OLFML3 expression and subcellular localization.

IRG1 encodes a decarboxylase that catalyzes the decarboxylation of *cis*-aconitate, leading to the production of itaconate. The immunomodulatory function of itaconate in LPS-induced metabolic reprogramming of macrophages has been known 48. The present study showed that OLFML3 could form intracellular complex with IRG1 and facilitate its mitochondrial localization (Figure 6, 7). Consistently, ablation of *Olfml3* gene significantly affected itaconate, succinate and ATP production (Figure 5). However, it must be noted that depletion of OLFML3 did not abolish the mitochondrial localization of IRG1 (Figure 7A, 7B). This observation suggested that there were alternative, OLFML3-independent mechanisms for IRG1 mitochondrial localization. In addition, *Irg1* overexpression could only partially restore MCP-1 expression in *Olfml3*^-/-^ macrophages. These results indicated that OLFML3 and IRG1 were mutually dispensable. In addition, we found that OLFML3 affected IRG1 protein levels though with a lesser extent than the effects on IRG1 subcellular localization. The protein-protein interaction network surrounding OLFML3 and IRG1 would be an interesting topic for future studies on OFLML3.

Related to the interaction between OLFML3 and IRG1 is the effects of metabolic reprogramming on LPS or bacterium-induced acute lung injury. Our results showed that OLFML3 promoted IRG1 mitochondrial localization and knockout of *Olfml3* reduced mitochondria-localized IRG1. Importantly, compared to wild-type (*Olfml3^+/+^*) macrophages, *Olfml3* knockout (*Olfml3^-/-^*) macrophages exhibited lower itaconate and succinate production (Figure 5A-D) as well as ATP level (Figure 5E). Meanwhile, compared to *Olfml3^+/+^* mice, *Olfml3^-/-^* mice had increased death rate and elevated inflammatory phenotype in response to LPS stimulation or PAO1 infection (Figure 1A-E). Therefore, the reduced ATP production and disrupted metabolic process in *Olfml3* knockout macrophages were correlated with exacerbated acute lung injury. These results were also consistent with the previous study, where ATP injection prior to LPS stimulation was shown to significantly attenuate LPS-induced inflammatory responses in mice 62.

In *Xenopus*, OLFML3, known as *Xenopus* ONT1, contributes to embryonic axial formation by acting as a scaffold protein. OLFML3 binds to chordin via OLF domain and to BMP1/Tolloid-class proteinases (B1TP) via CC domain 5. The present study suggested that OLFML3 in mammalian cells resembled such scaffold function by interacting with IRG1 via its OLF domain and with AIFM1, a known mitochondrial transport protein, via its CC domain (Figure 7F). In addition to the identification of intracellular tertiary complex of IRG1-OLFML3-AIFM1, our study also suggested that the formation of OLFML3-IRG1 complex was dependent on the presence of AIFM1 (Supplemental Figure 6D). Interestingly, it was also found that IRG1 itself could form a high-molecular weight complex in the absence of OLF domain of OLFML3 (Supplemental Figure 4C) or AIFM1 (Supplemental Figure 6D), which possibly arose from the oligomerization of IRG1 protein 41.

In this study, unaddressed questions include effects of post-translational modification (PTM) and epigenetic modulation on OLFML3 and IRG1. It is known that OLFML3 has two glycosylation sites at positions 177 and 248 13, the function of which was largely unknown. The crystal structure of IRG1 has been reported to have has open and closed conformations 41, 42, the switching between which is closely related to its activity and may be dependent on PTMs. It has been also shown that in *Caenorhabditis elegans*, S-adenosylmethionine (SAM) treatment can enhance innate immunity by increasing the H3K4me3 of *irg-1* gene 63. Thus, it would be interesting to investigate the effects of PTMs and epigenetics on the immunomodulatory functions of OLFML3 and IRG1 in human.

**Figure 8:**
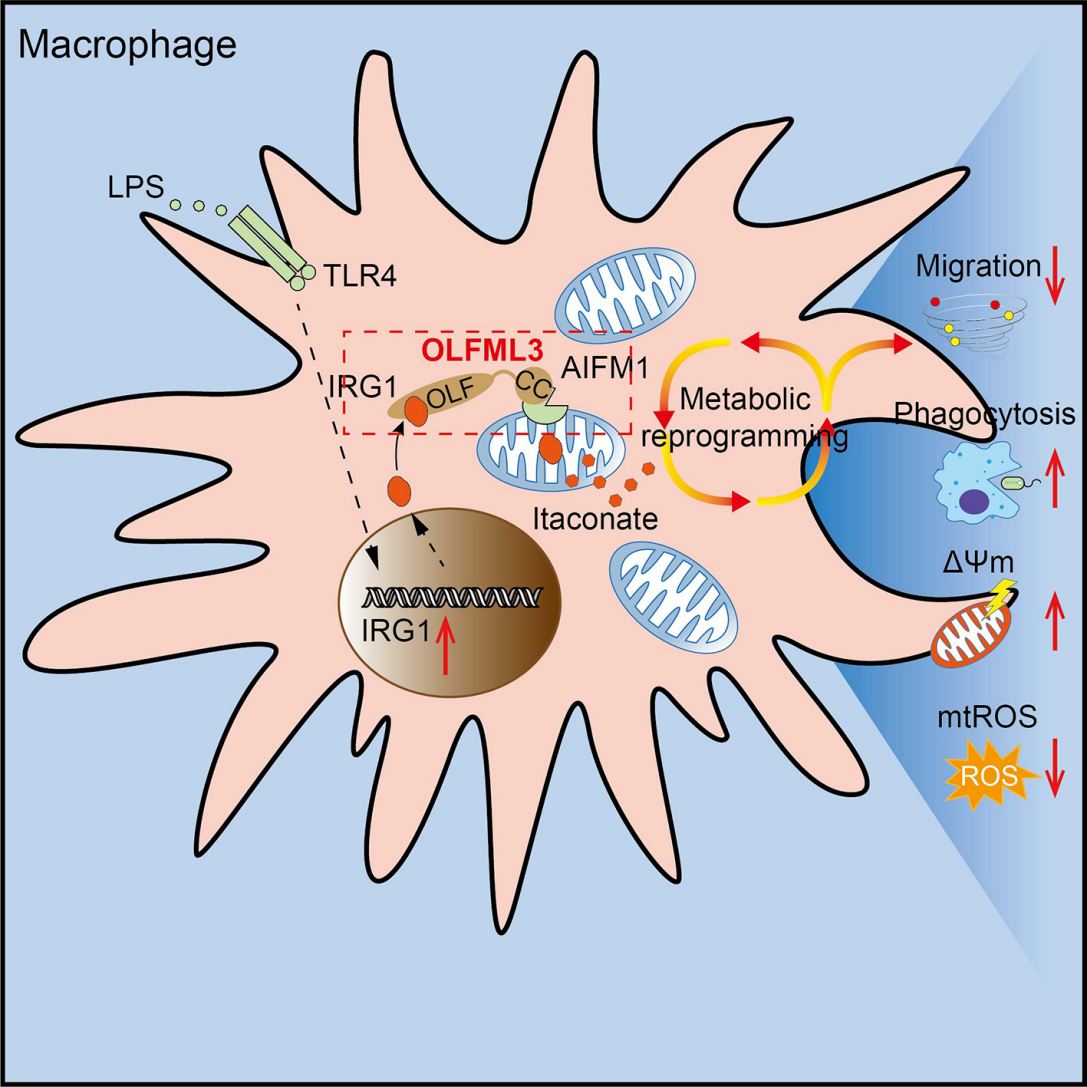
**Schematic mechanisms of the modulatory function of OLFML3 in macrophages.** OLFML3 facilitates IRG1mitochondrial localization via a mitochondrial transporter protein AIFM1 in macrophages. Depletion of OLFML3 in LPS-induced macrophages leads to decreased IRG1 mitochondrial localization and itaconate metabolic processes, and causes MMP loss and mtROS over-production, which in turn suppresses macrophage phagocytosis and migration and results in aggravated ALI.

In conclusion, our study discovered that OLFML3 could suppress the inflammation and severity of mice in LPS-induced ALI model. This function arose from OLFML3-facilitated macrophage phagocytosis and migration, which was mechanistically attributed to OLFML3-mediated IRG1 mitochondrial localization and prevention of MMP loss and mtROS over-production (Figure 8).

## Supplementary Material

Supplementary figures, movie and dataset legends and description.

Supplementary dataset 1.

Supplementary dataset 2.

Supplementary dataset 3.

Supplementary movie 1.

Supplementary movie 2.

Supplementary movie 3.

Supplementary source data.

### Data availability

Plasmids and cell lines generated in this study will be made available on request, but we may require a payment and/or a completed Materials Transfer Agreement if there is potential for commercial application. The RNA-seq data reported in this paper have been deposited on GEO DataSets under accession code GSE289765. A web page to reproduce RNA-seq findings is made accessible at http://www.genetictargets.com/OLFML3.

## Abbreviations

OLFML3: olfactomedin-like protein 3
OLF: olfactomedin
CC domain: coiled-coil region
OLF domain: OLF-like domain
OLFM4: olfactomedin 4
LPS: lipopolysaccharide
ALI: acute lung injury
IRG1: immunoresponsive gene 1
BMDMs: bone marrow-derived macrophages
MMP/∆Ψm: mitochondrial membrane potential
mtROS: mitochondrial reactive oxygen species
AIFM1: apoptosis inducing factor mitochondria associated 1
B1TP: BMP1/Tolloid-class proteinase
RV: rhinovirus
PAMPs: pathogen-associated molecular patterns
W/D: wet/dry
BALF: bronchoalveolar lavage fluid
DEGs: differentially expressed genes
GO: Gene ontology
MCP-1: monocyte chemoattractant protein-1
ΔSP-OLFML3: protein deficient of signal peptide
OFP: orange fluorescent protein
