# Supplementary material for: OLFML3 Promotes IRG1 Mitochondrial Localization and Modulates Mitochondrial Function in Macrophages
Source: Int J Biol Sci. 2025 Feb 26;21(5):2275–95. doi: 10.7150/ijbs.103859 (PMC11900800; doi:10.7150/ijbs.103859)
Supplement: Supplementary file 1 — Supplementary figures, movie and dataset legends and description. [file ijbsv21p2275s1.pdf]

Supplemental Materials

Table of Contents

1. **Supplemental Figures**
2. **Supplemental Movies**
3. **Supplemental Dataset**

Supplemental Figures

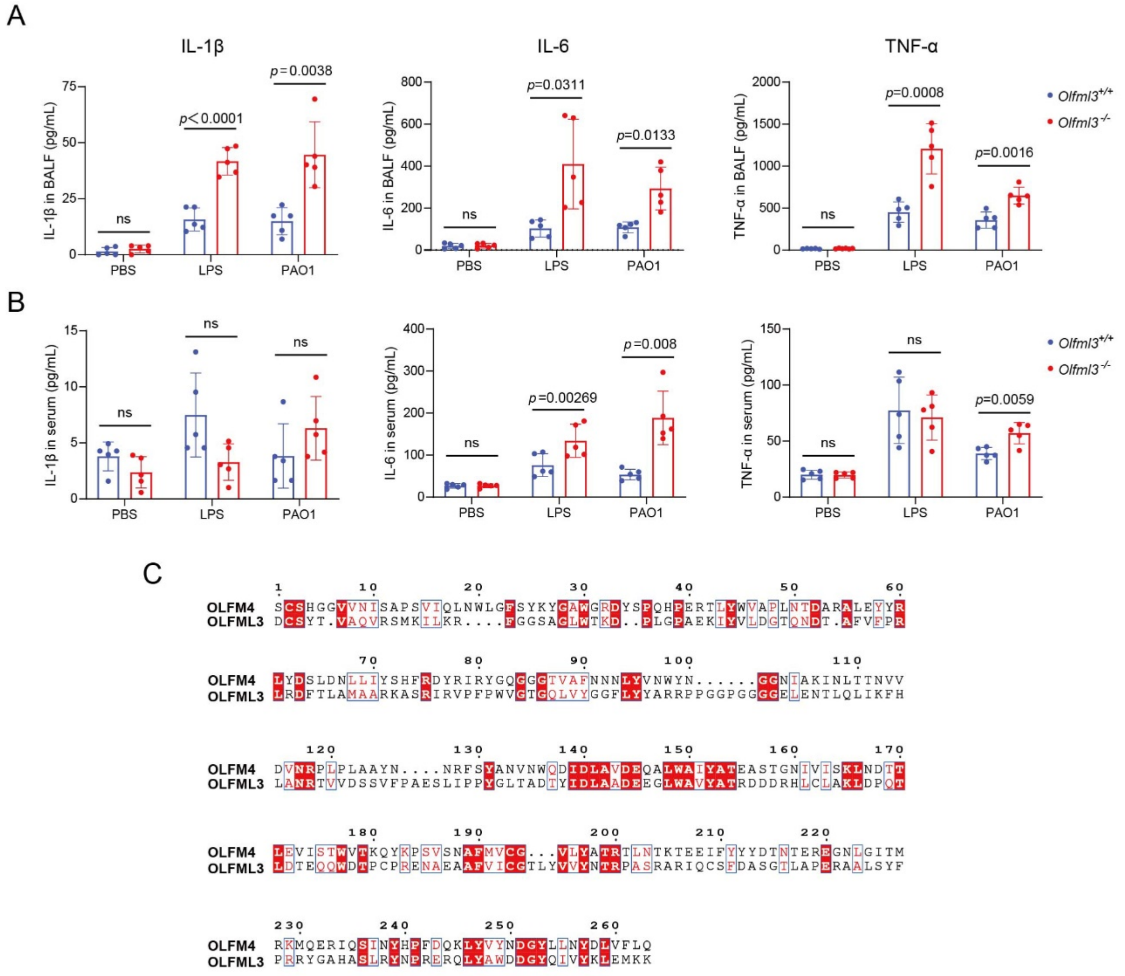

Fig. S1. Analysis of the in vivo function of OLFML3 in LPS- or PAO1-induced lung injury model in mice. (A, B) Analysis of the expression of pro-inflammatory cytokines (IL-1β, IL-6, and TNF-α) in BALF (A) and serum (B) by ELISA. LPS (10 mg/kg) or PAO1 (2×10^6 CFU) is intranasally instilled to mice (n = 5 per group). The data are presented as mean ± SD of three independent experiments and significant difference analyzed by two-tailed Student's t test. ns, not significant. (C) Alignment of the OLF domains of murine OLFM4 and OLFML3. Amino acid sequence alignment is conducted by ClustalW. The alignment results are visualized by ENDscript/ESPript website.

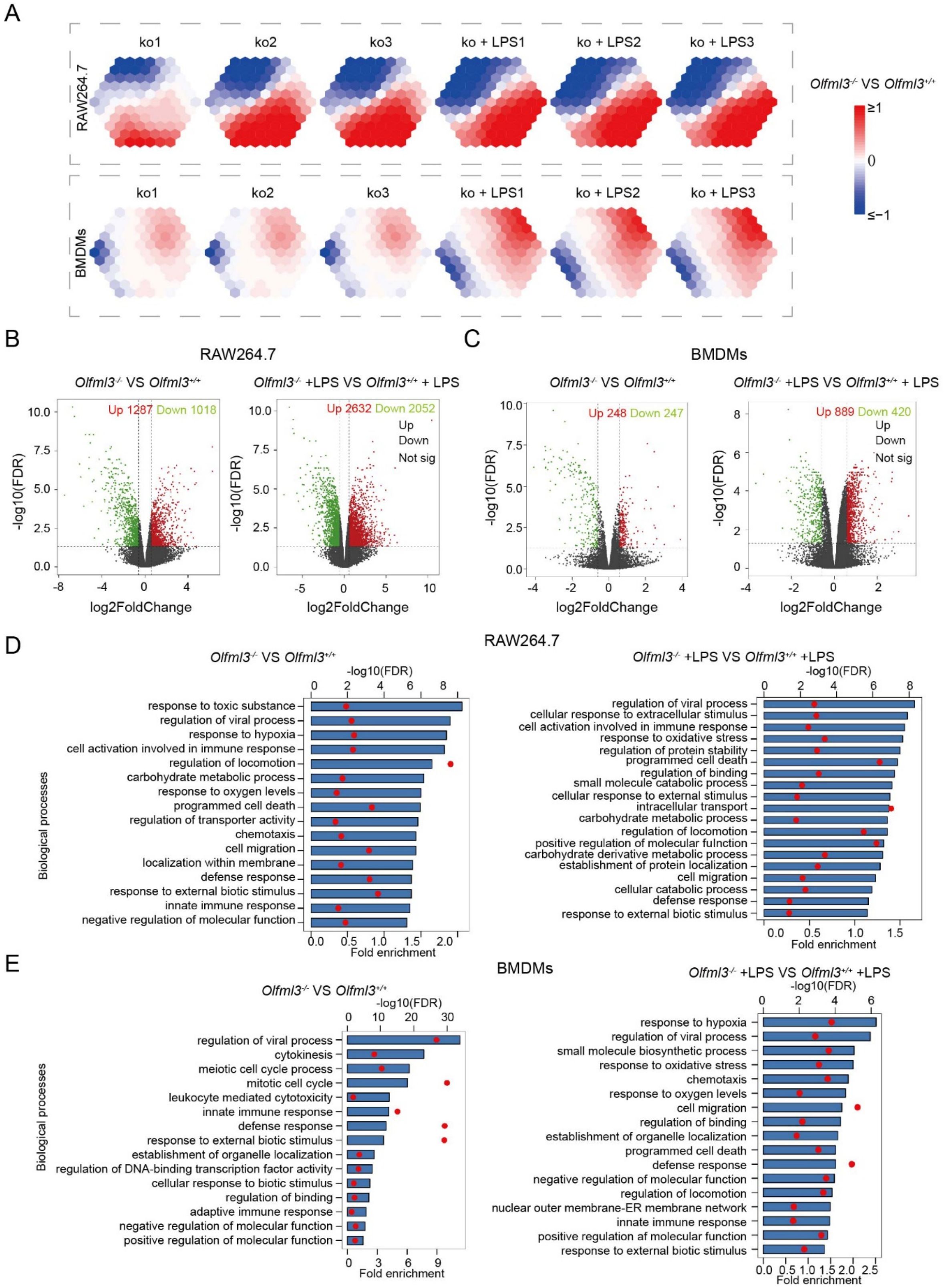

Fig. S2. RNA-Seq analyses of the effects of *Olfml3* knockout in RAW264.7 cells and BMDMs in the absence and presence of LPS stimulation. (A) Gene clustering

and sample correlation as shown by supra-hexagonal maps. The genes with similar expression pattern in response to *Olfml3* knockout are clustered in the same hexagons and hexagons with similar fold change values are clustered as neighbours. The colors indicate normalized fold change values. (B, C) Volcano plot showing differentially expressed genes in RAW264.7 (B) and BMDMs (C). DEGs with adjusted p value of more than 0.05 and fold change of more than 1.5 are highlighted in red (upregulation) and green (downregulation). (D, E) The top biological processes of DEGs in RAW264.7 cells (D) and BMDMs (E). The bars and dots dictate fold enrichment and -log values of false discovery rate (FDR) respectively. LPS: 100 ng/mL. The RNA-Seq data are from three biological replicates. *Olfml3*$^{-/-}$ RAW264.7 is a single clone isolated from CRISPR-generated knockout cells. *Olfml3*$^{-/-}$ BMDMs are acquired from the tibial and femoral bones of *Olfml3*$^{-/-}$ mice.

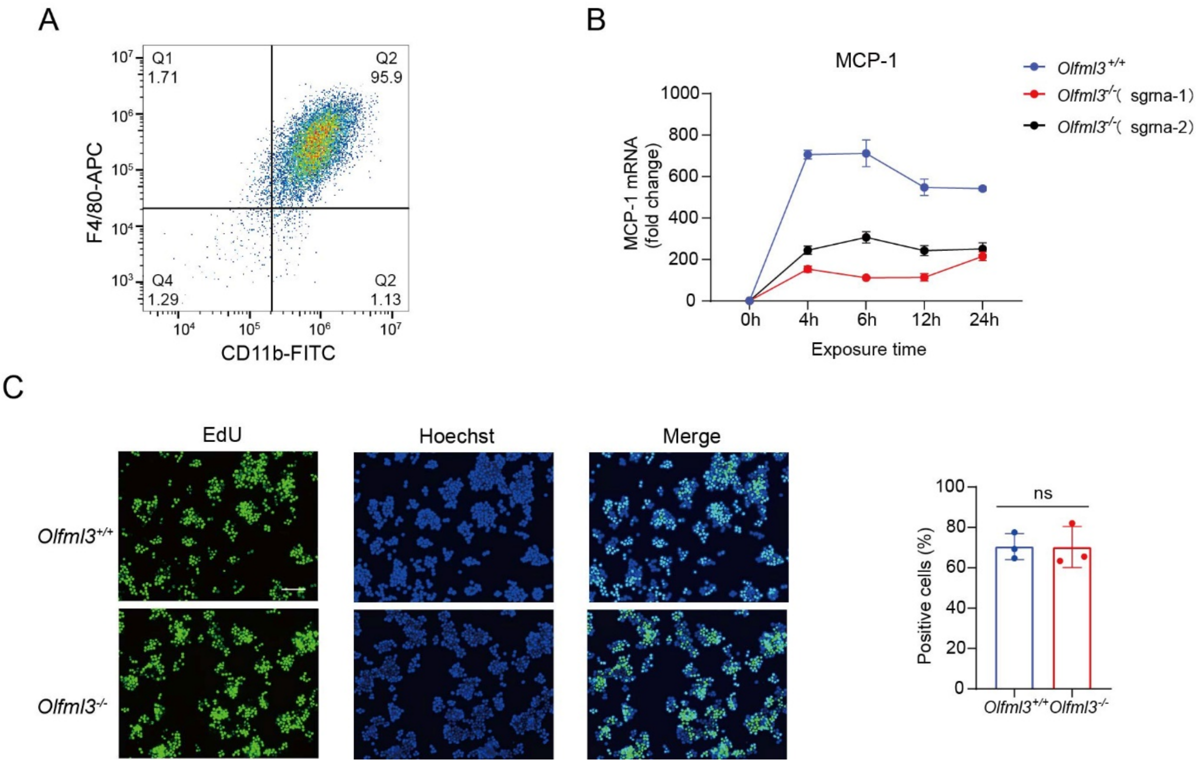

Fig. S3. Analysis of the effects of *Olfml3* knockout in RAW264.7 cells and BMDMs (related to Fig. 2). (A) Flow cytometry characterization of isolated BMDMs at 7 days after differentiation. F4/80 and CD11b are used as markers of BMDMs. (B) Analysis of the effects of *Olfml3* knockout on MCP-1 mRNA expression in LPS (100 ng/mL)-stimulated RAW264.7 cells over a course of 24 h. Two single clones are generated using different sgRNAs to limit the effects of off-target targeting. (C) Proliferation of wild-type and *Olfml3*$^{-/-}$ RAW264.7, as measured by EdU assay.

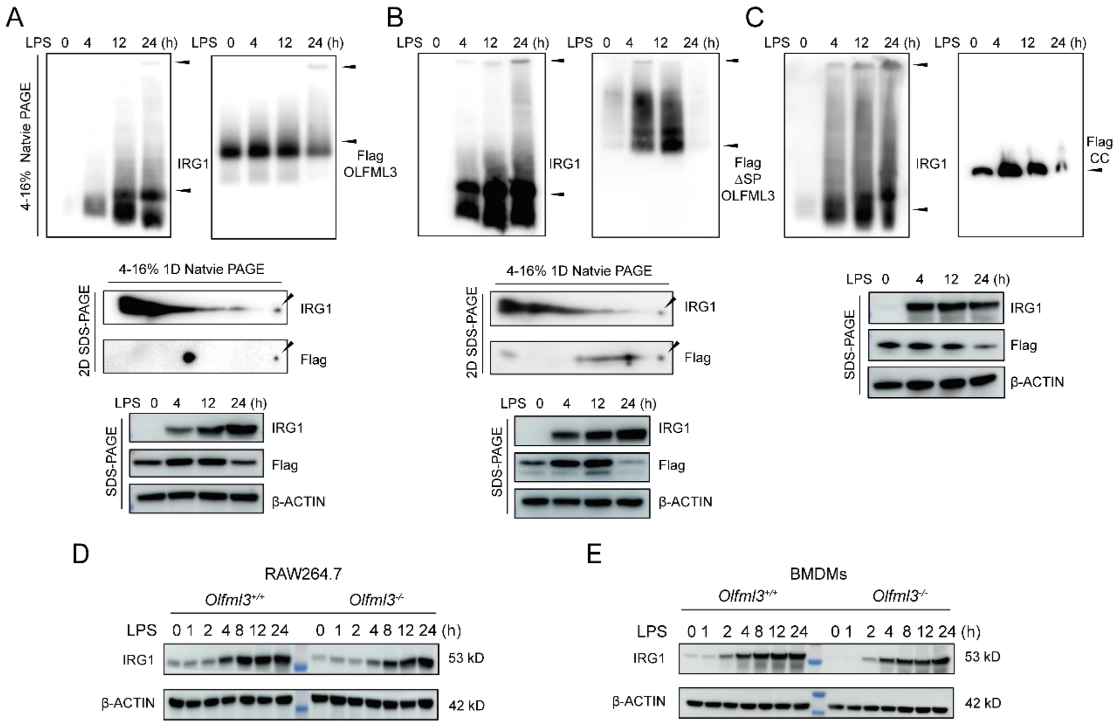

Fig. S4. Visualization of LPS-induced formation of intracellular OLFML3-IRG1 complex using 1D native PAGE and 2D SDS-PAGE. (A-C) The interactions between endogenous IRG1 and OLFML3 transgene in RAW264.7 cells stably expressing OLFML3-Myc/Flag (A), ΔSP-OLFML3-Myc/Flag (B) or CC domain-Myc/Flag (C) constructs. The cells are stimulated by 100 ng/mL LPS over a course of 24 h. The OLFML3-IRG1 complex is visualized both on 1D native PAGE (top) and on 2D PAGE (middle) with sequential runs of native PAGE (horizontal) and denaturing SDS-PAGE (vertical). OLFML3 and IRG1 are immunoblotted using IRG1 and Flag antibodies respectively. (D, E) Evaluation of the effects of *Olfml3* knockout on IRG1 expression in RAW264.7 cells (D) and BMDMs (E).

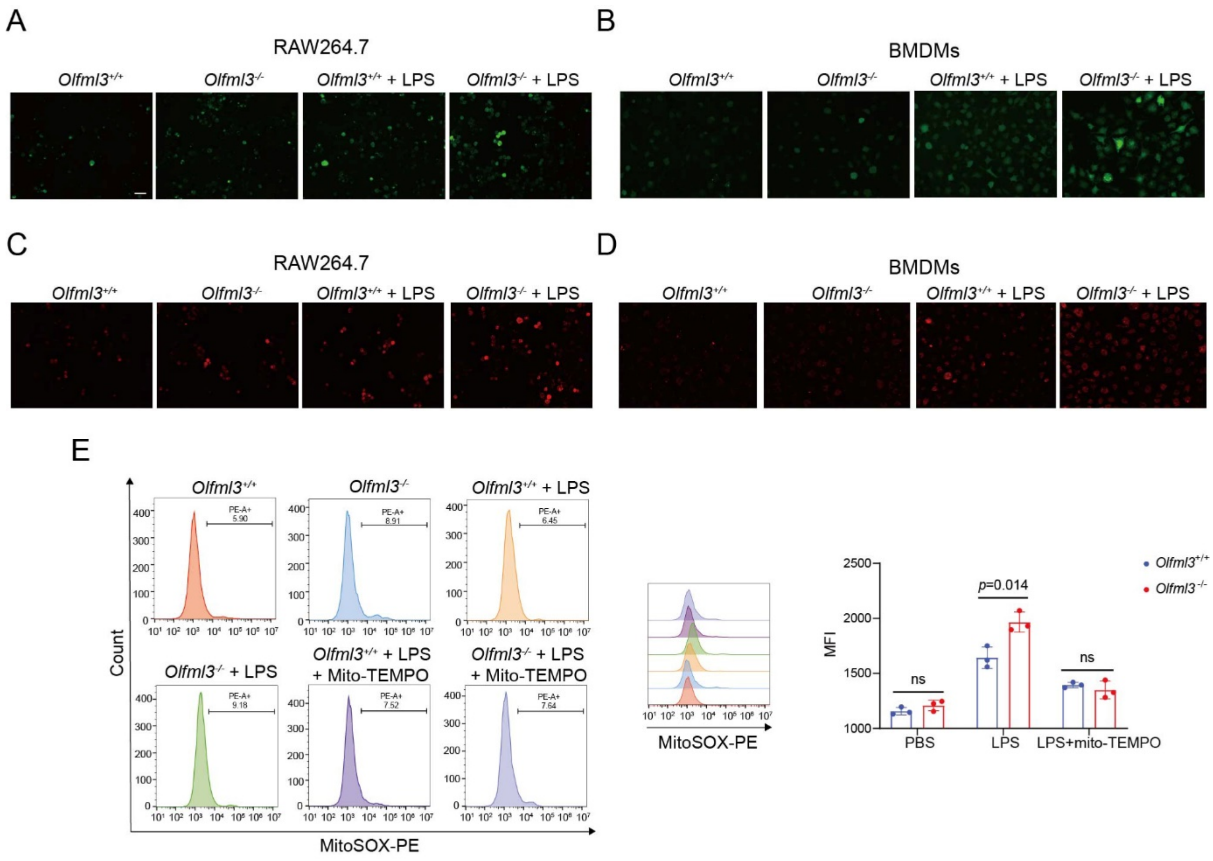

Fig. S5. Evaluation of the effects of OLFML3 on MMP and ROS production of macrophages. (A, B) Evaluation of the effects of OLFML3 on LPS-stimulated total cellular ROS production in RAW264.7 (A) and BMDMs (B), analyzed by immunofluorescence. Total ROS is detected using DCFH-DA fluorescent probe. (C, D) Evaluation of the effects of OLFML3 on LPS-stimulated mtROS production in RAW264.7 (C) and BMDMs (D), analyzed by immunofluorescence. Mitochondrial ROS is detected using MitoSOX fluorescent probe. (E) Analysis of the effects of Mito-TEMPO (500 μM, 2 h pre-treatment) on *Olfml3* knockout-induced upregulation of mtROS in RAW264.7 cells using MitoSOX fluorescence probe. MFI, median fluorescent intensity. Cells are collected at 12 h post 100 ng/mL LPS stimulation.

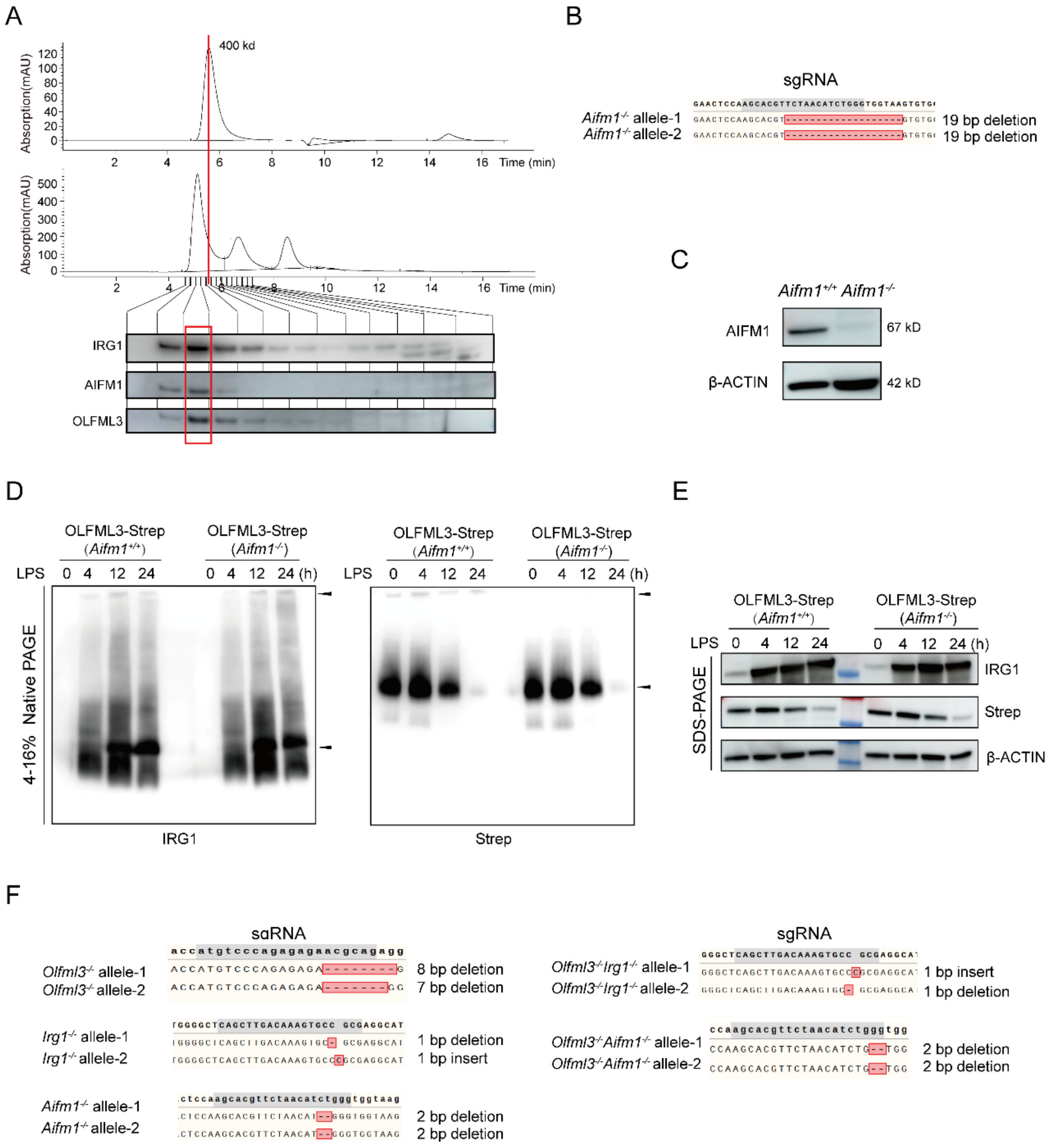

Fig. S6. OLFML3-IRG1 complex formation is dependent on mitochondrial transport protein AIFM1. (A) The absorption at 280 nm of purified Cascade-crRNA complex (405 kD) as a marker and of cell supernatants of LPS-treated RAW264.7 cells. The 13 fractions of the elution from 4.607 min to 7.007 min were resolved on SDS-PAGE and proteins detected by WB. (B, C) Characterization of an *Aifm1* knockout single clone of RAW264.7 cells with stable expression of OLFML3-Strep by genomic analyses of mutated alleles (B) and by WB (C). (D, E) Evaluation of the effects of *Aifm1* knockout on the formation of OLFML3-IRG1 complex in RAW264.7 cells stably expressing OLFML3-Strep. The cells are stimulated with 100 ng/mL LPS for indicated durations, and the total cellular proteins resolved on native PAGE (D) or SDS-PAGE

(E), and immunoblotted with IRG1 and Strep antibodies respectively as indicated. (F) Characterization of mutated gene alleles of *Olfml3*, *Irg1* and *Aifm1* single knockout and *Olfml3*$^{-/-}$*Irg1*$^{-/-}$ and *Olfml3*$^{-/-}$*Aifm1*$^{-/-}$ double knockout RAW264.7 cells.

Supplemental Movies

Movie S1. 3D-SIM acquisitions of the HeLa cells stably expressing OLFML3-OFP (red) and IRG1-GFP (green) (attached as a separate file).

Movie S2. Live-cell fluorescence time-lapse microscopy of the HeLa cells stably expressing OLFML3-OFP (red), IRG1-GFP (green) and Mitotracker (blue) after treatment with 1 μg LPS for 12 h (attached as a separate file).

Movie S3. Live-cell fluorescence time-lapse microscopy of the HeLa cells stably expressing OLFML3-OFP (red), IRG1-GFP (green) and Mitotracker (blue) without LPS (attached as a separate file).

Supplemental Dataset

Dataset S1. Results of RNA-seq

Sheet 1-4. The genes in the Volcano diagram as shown in Supplemental Figure 2B, 2C.

Sheet 5-8. The genes and pathways in GOBP as shown in Supplemental Figure 2D, 2E.

Sheet 9. The genes in the RNA-seq-Self-organizing map as shown in Supplemental Figure 2A.

Dataset S2. Results of CoIP-MS

Sheet 1. Proteins identified in Olfml3-myc-flag IP-MS as shown in Figure 3A.

Sheet 2. Proteins identified in Olfml3-Strep IP-MS as shown in Figure 7D.

Dataset S3. Sequences of primers, gRNAs and genes used in this study

Sheet 1. The sequence of primers used for qPCR.

Sheet 2. The sequence of gRNAs used for gene knockout.

Sheet 3. The sequence of primers used for genotyping or sequencing.

Sheet 4. DNA sequences of recombinant gene.
